# Antivascular and antitumor properties of the tubulin-binding chalcone TUB091

**DOI:** 10.18632/oncotarget.9527

**Published:** 2016-05-20

**Authors:** María-Dolores Canela, Sam Noppen, Oskía Bueno, Andrea E. Prota, Katja Bargsten, Gonzalo Sáez-Calvo, María-Luisa Jimeno, Mohammed Benkheil, Domenico Ribatti, Sonsoles Velázquez, María-José Camarasa, J. Fernando Díaz, Michel O. Steinmetz, Eva-María Priego, María-Jesús Pérez-Pérez, Sandra Liekens

**Affiliations:** ^1^ Instituto de Química Médica (IQM-CSIC), Madrid, Spain; ^2^ KU Leuven – University of Leuven, Rega Institute for Medical Research, Laboratory of Virology and Chemotherapy, Leuven, Belgium; ^3^ Laboratory of Biomolecular Research, Department of Biology and Chemistry, Paul Scherrer Institut, Villigen, Switzerland; ^4^ Centro de Investigaciones Biológicas (CIB-CSIC), Madrid, Spain; ^5^ Centro de Química Orgánica Lora-Tamayo (CENQUIOR-CSIC), Madrid, Spain; ^6^ Department of Basic Medical Sciences, Neurosciences and Sensory Organs, University of Bari Medical School, National Cancer Institute “Giavanni Paolo II”, Bari, Italy

**Keywords:** cancer, drug research, tubulin, vascular-disrupting

## Abstract

We investigated the microtubule-destabilizing, vascular-targeting, anti-tumor and anti-metastatic activities of a new series of chalcones, whose prototype compound is (E)-3-(3’’-amino-4’’-methoxyphenyl)-1-(5’-methoxy-3’,4’-methylendioxyphenyl)-2-methylprop-2-en-1-one (TUB091). X-ray crystallography showed that these chalcones bind to the colchicine site of tubulin and therefore prevent the curved-to-straight structural transition of tubulin, which is required for microtubule formation. Accordingly, TUB091 inhibited cancer and endothelial cell growth, induced G2/M phase arrest and apoptosis at 1-10 nM. In addition, TUB091 displayed vascular disrupting effects *in vitro* and in the chicken chorioallantoic membrane (CAM) assay at low nanomolar concentrations. A water-soluble L-Lys-L-Pro derivative of TUB091 (i.e. TUB099) showed potent antitumor activity in melanoma and breast cancer xenograft models by causing rapid intratumoral vascular shutdown and massive tumor necrosis. TUB099 also displayed anti-metastatic activity similar to that of combretastatin A4-phosphate. Our data indicate that this novel class of chalcones represents interesting lead molecules for the design of vascular disrupting agents (VDAs). Moreover, we provide evidence that our prodrug approach may be valuable for the development of anti-cancer drugs.

## INTRODUCTION

Cancer is a leading cause of death worldwide and its occurrence is expected to double in the following decades [1]. Resistance and lack of efficacy associated with current antineoplastic treatments demand the design of novel anticancer drugs [2]. Vascular Disrupting Agents (VDAs) constitute an innovative approach in anticancer therapy due to their specific mechanism of action, which is complementary to other existing therapies [3–5]. VDAs act directly and selectively at the tumor endothelium inducing detrimental morphological and functional changes. Consequently, blood flow inside the tumor is quickly and dramatically decreased resulting in massive necrosis due to the hypoxic conditions [6]. The selectivity of VDAs for tumor endothelium versus physiological vessels lies in the crucial differences between these vessels [4, 6]. Tumor vessels are characterized by a higher proliferation rate and the lack of pericytes and a proper basement membrane, which makes them more fragile and tortuous than physiological vessels [6, 7]. Overall, this results in increased vascular permeability and a higher resistance to blood flow, making the tumor vasculature more sensitive to any variation in perfusion pressure [8, 9].

**Figure 1:**
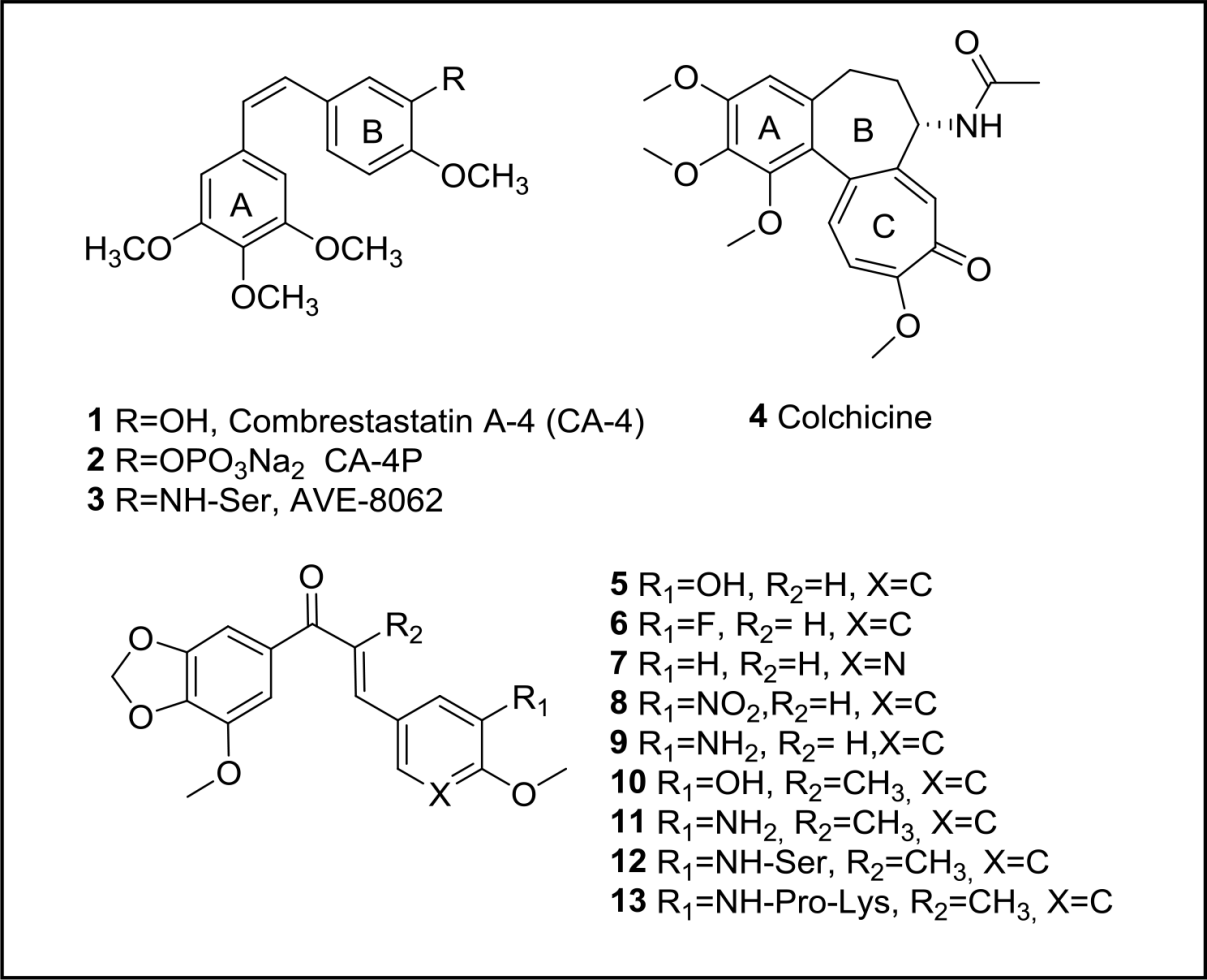
Structural formulae of reference compounds and newly synthesized chalcones

The best studied VDAs are microtubule-destabilizing agents that bind the αβ-tubulin dimer at the colchicine-binding site. The most representative compounds are combretastatin A4 (CA-4, 1, Figure 1) and its prodrugs (CA-4 phosphate (2) or the serine derivative AVE-8062 (3)) [10, 11]. In general, colchicine-site binders, when affecting tumor endothelial cells, block tubulin assembly into microtubules, which in turn induces a large cascade of events, such as actin stress fiber contraction and the subsequent activation of Rho kinases. Consequently, endothelial cells round up and lose their connectivity, leading to vascular collapse, tumor hypoxia and finally hemorrhagic tumor necrosis [12]. In addition, colchicine-site binders are also able to behave as antimitotic agents [13]. This antimitotic effect is closely related to the importance of the microtubule cytoskeleton in the formation of the mitotic spindle during cell division [14]. As a consequence, several colchicine-site binders are currently being evaluated clinically in different combination regimens for the treatment of a variety of solid tumors [15].

Besides colchicine (4, Figure 1) and CA-4, natural products continue to be a source of microtubule-destabilizing agents binding at the colchicine-site. We have centered our attention at those compounds that include a dioxolane group at ring A, such as combretastatin A2 [16], polygamain [17] or cornigerin [13]. Another interesting group of natural product-inspired compounds binding at the colchicine-site are chalcones, such as MDL-27048, a synthetic chalcone with significant antitumor effects *in vitro* and *in vivo* [18]. Other chalcones have been designed and synthesized with the purpose to enhance the chemical stability of CA-4 [11, 19].

Here, we synthesized new colchicine-site binders with a chalcone scaffold, along with a dioxolane motif at ring A, according to its abundant presence in natural colchicine-site ligands. X-ray crystallography was performed to gain detailed insight into the binding mode of this family of chalcones. The anti-proliferative and anti-vascular properties of the compounds were determined and a prodrug strategy was applied in order to improve the water solubility of the most active compound (TUB091). This prodrug (TUB099) was shown to possess potent anti-tumor and vascular-disrupting activities in primary tumor models and proved as potent as the reference compound CA-4P in inhibiting spontaneous metastasis induced by human breast cancer MDA-MB-231 cells.

## RESULTS

### Synthesis of the chalcones

The synthesis of the chalcones involved an aldol condensation between a phenone fused to a dioxolane ring (A ring) and the corresponding benzaldehyde (ring B). First, we envisioned the synthesis of the unsubstituted chalcones at the α position of the α,β-unsaturated ketone (Supplementary Scheme 1). This resulted in chalcones 5–9 (Figure 1) of which 5 and 9 with a hydroxyl or an amine group at position 3 of ring B, respectively, showed the best growth-inhibitory activity (Table 1, see further). Based on these data the second series of chalcones (Supplementary Scheme 1), incorporating a methyl group at the α position of the α,β-unsaturated ketone, contained only those compounds whose B-ring had the same substitution as 5 and 9, thus providing compounds 10 (TUB092) and 11 (TUB091) (Figure 1).

**Table 1:** Growth-inhibitory activity of reference compounds and newly synthesized chalcones

	Endothelial Cells		Tumor Cells		
Compound	IC_50_ (μM)		IC_50_ (μM)		
	HMEC-1	BAEC	B16-F10.luc2	CEM	HeLa
CA-4P (2)	0.0029 ± 0.0001	0.0039 ± 0.0002	0.0032 ± 0.0002	0.011 ± 0.001	0.013 ± 0.001
Colchicine (4)	0.0038 ± 0.0011	0.0069 ± 0.0008	0.021 ± 0.002	0.013 ± 0.001	0.0087 ± 0.0003
5	0.86 ± 0.90	0.040 ± 0.009	0.25 ± 0.18	0.67 ± 0.67	0.35 ± 0.26
6	1.6 ± 0.0	1.9 ± 0.3	7.8 ± 3.7	23 ± 0	5.8 ± 5.0
7	5.4 ± 0.7	4.3 ± 1.5	8.7 ± 1.6	28 ± 8	16 ± 2
8	2.1 ± 0.1	2.9 ± 2.5	12 ± 4	10 ± 0	7.2 ± 0.1
9	0.015 ± 0.007	0.0095 ± 0.0078	0.039 ± 0.023	0.080 ± 0.000	0.22 ± 0.09
TUB092 (10)	0.013 ± 0.002	0.0036 ± 0.0003	0.012 ± 0.001	0.0027 ± 0.0012	0.013 ± 0.004
TUB091 (11)	0.0023 ± 0.0005	0.0029 ± 0.0005	0.0031 ± 0.0001	0.0010 ± 0.0007	0.0039 ± 0.0033

Growth-inhibitory activity is presented as IC_50_, i.e. concentration that reduces cell growth by 50%. HMEC-1: human microvascular endothelial cell line-1; BAEC: bovine aortic endothelial cells; B16-F10.luc2: mouse melanoma cells expressing firefly luciferase 2; Cem: human lymphocytic leukemia cells; HeLa, human cervical carcinoma cells. Data are mean ± SD.

### Growth-inhibitory activity of the chalcones

The synthesized compounds were first evaluated for their growth-inhibitory activity in cancer and endothelial cell lines (Table 1). As reference compounds CA-4P (2) and colchicine (4) were included. Among unsubstituted chalcones at the α-position (5–9), compounds 5 and 9, with a 3-hydroxy or 3-amine group at ring B, respectively, showed 50% inhibitory values (IC_50_) in the sub-micromolar range. The introduction of a methyl group at the α-position of the double bond (compounds 10 and 11) significantly improved the growth-inhibitory activity. Importantly, the aminochalcone 11 (TUB091), with IC_50_ values between 1 and 4 nM, proved to be even more active than the reference compounds in all cell lines tested (Table 1). CA-4P as well as TUB091 also inhibited the growth of normal fibroblasts and peripheral blood mononuclear cells (PBMC) in the nanomolar range (Supplementary Table 1). However, it should be noted that, whereas the compounds were toxic at micromolar concentrations in tumor and endothelial cells (see further), they were not toxic up to 100 μM in PBMCs as evidenced by the fact that a slight growth of these cells was still observed at 100 μM of compound.

### Chalcones bind to the colchicine-site of tubulin

**Figure 2:**
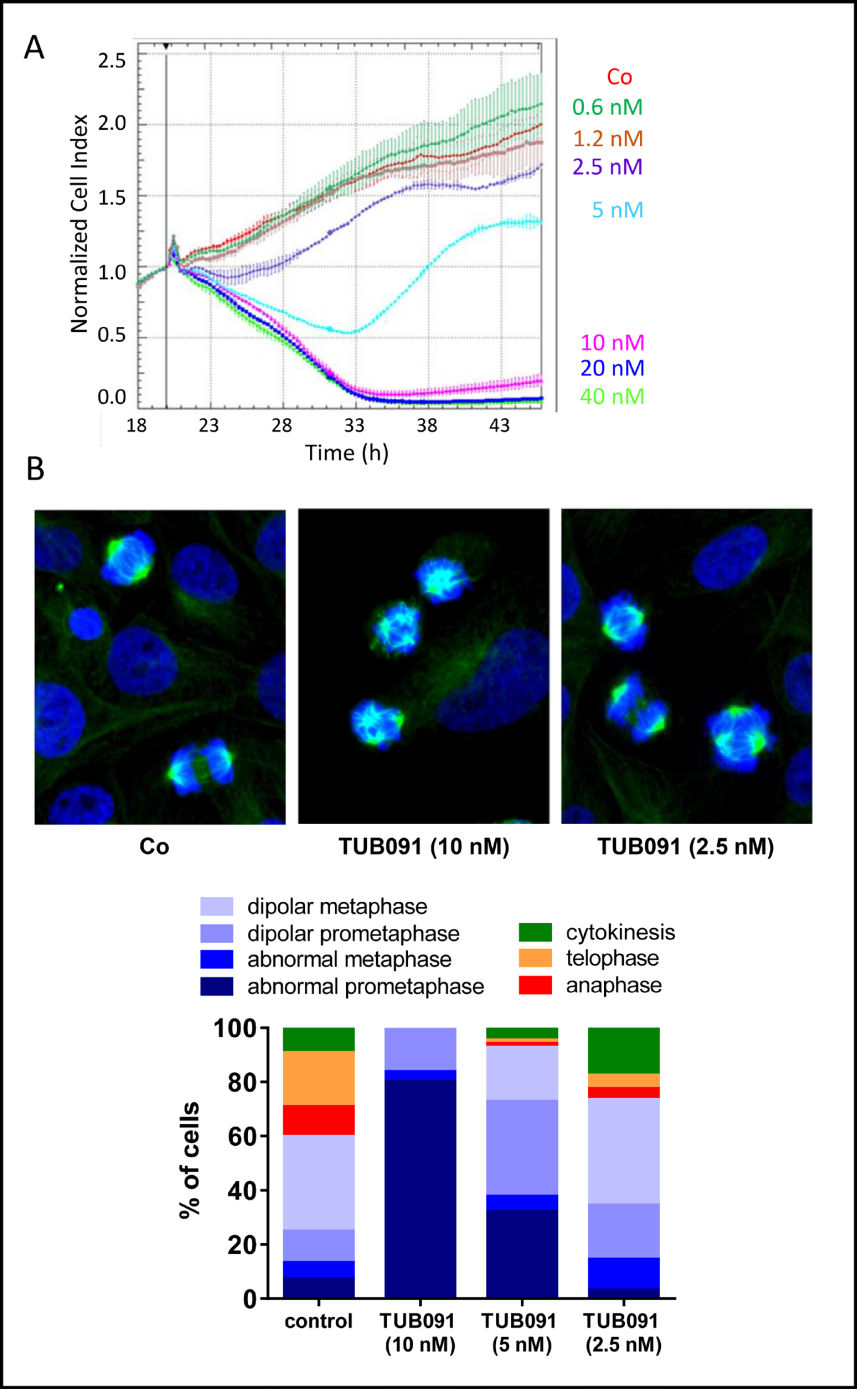
Tubulin-binding activity of chalcones (**A**) Growth curve of MDA-MB-231 cells in the presence of different concentrations of TUB091. The graph presents the normalized cell index (based on real-time impedance measurements) from 18 to 46 h after cell seeding. Compound was added 20 h after cell seeding (vertical line). Fast reduction in cell index with recovery of surviving cells after 14 h treatment (as seen at 5 nM) is specific for tubulin-binding compounds. (**B**) MDA-MB-231 cells were treated for 15 h with different concentrations of TUB091, fixed and stained with anti-β-tubulin antibody (green) to visualize the microtubules and Hoechst for DNA (blue). Cell cycle phase and spindle organization were investigated in individual cells (minimum 100 per condition) and quantified based on spindle morphology.

Using impedance-based monitoring of cell growth, we found a time- and dose-dependent anti-mitotic signature in MDA-MB-231 breast cancer cells treated with TUB091. At concentrations higher than 5 nM, TUB091 caused a rapid and continuous decrease in cell index, indicative of reduced cell adhesion and/or toxicity. Using lower concentrations of TUB091, a decrease in cell index during the first 14 h and subsequent recovery during the next 24–48 h was observed, typical for tubulin-targeting agents [20] (Figure 2A). Accordingly, staining of the microtubules in MDA-MB-231 cells after 15 h treatment with TUB091 resulted in the appearance of aberrant spindles. At 10 and 5 nM of TUB091, highly irregular and multipolar spindles were noticed, leading to a high number of cells in (pro)metaphase incapable of proceeding to telophase (Figure 2B), while at 2.5 nM the majority of cells underwent proper cell division.

The affinity of the compounds for bovine αβ-tubulin was studied by competition experiments with R-PT, whose binding to tubulin is characterized by fluorescence emission (K_a_ of 3.2 × 10^6^ M^−1^) [21, 22]. The binding constant obtained for compound 10 by R-PT displacement was (1.3 ± 0.2) × 10^7^ M^−1^, similar to the reported value for colchicine (1.2 × 10^7^ M^−1^ at 37°C). Since the binding of TUB091 to tubulin resulted in a change of the fluorescence emission with the same excitation wavelength of R-PT, the binding constant of TUB091 to tubulin was determined by displacement of the fluorescence of this compound by 10. TUB091 showed a K_a_ of (1.1 ± 0.1) × 10^7^ M^–1^ similar to that of 10 and colchicine.

**Figure 3:**
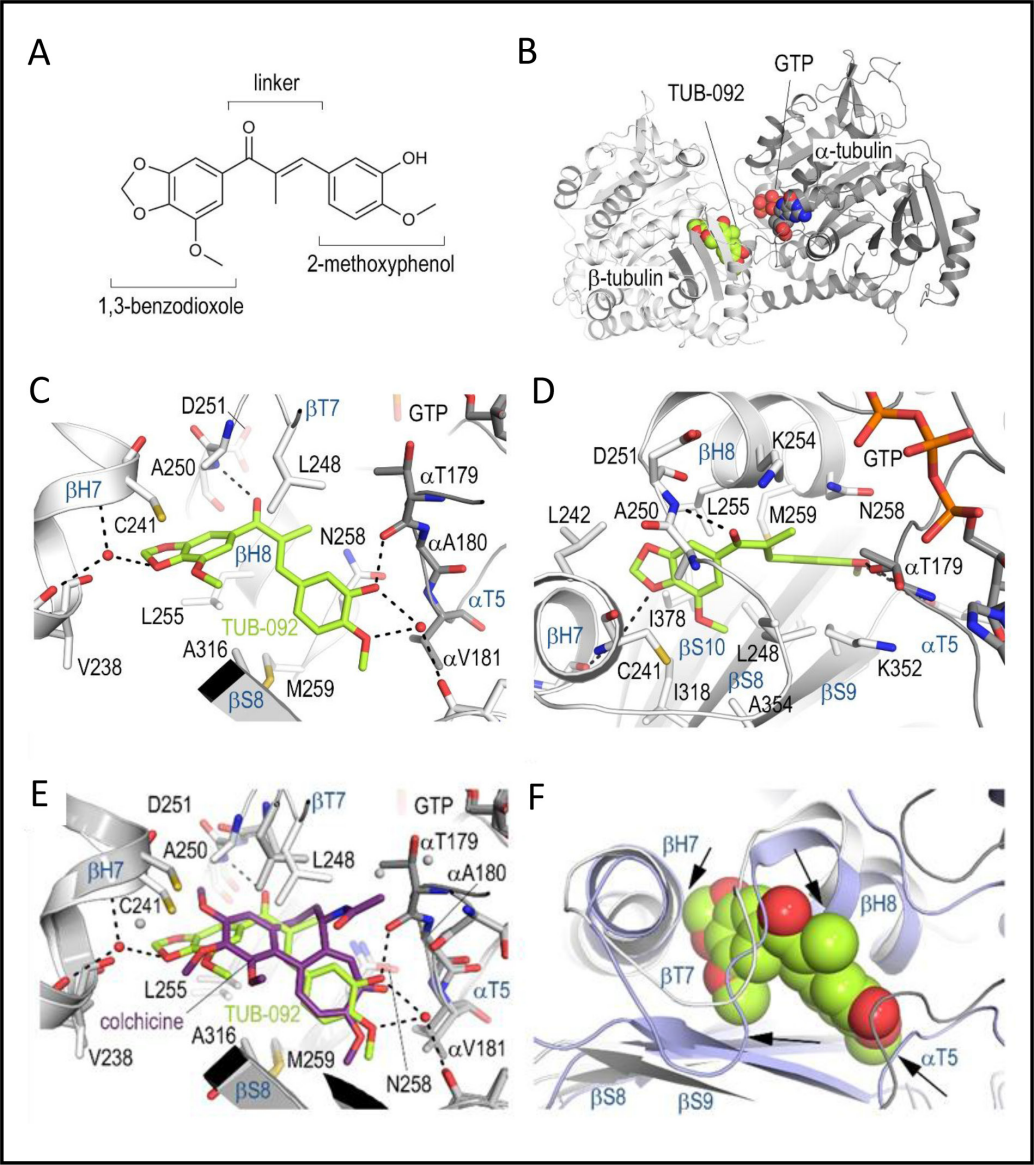
X-ray analysis of the chalcone-tubulin complex (**A**) Chemical structure of TUB092. (**B**) Overall view of the complex formed between αβ-tubulin and TUB092. α- and β-tubulin are in dark and light grey, respectively. (**C**, **D**). Close-up views of the interaction network observed between TUB092 (green) and tubulin (gray). Interacting residues of tubulin are shown in stick representation and are labeled. Oxygen and nitrogen atoms are colored red and blue, respectively; carbon atoms are in green (TUB092) or gray (tubulin). Hydrogen bonds are depicted as black broken lines. Secondary structure elements of tubulin are labeled in blue. (D) 90° rotation of C. (E-F) Comparison of TUB092 and colchicine tubulin-binding modes. (**E**) Superimposition of the tubulin–TUB092 (dark gray ribbons) and tubulin–colchicine (PDB ID 4O2B, light gray ribbons) structures. TUB092 and colchicine are in green and violet-purple stick representation, respectively. Water molecules are displayed as red (TUB092 structure) and grey (colchicine structure) spheres, respectively. (**F**) Superimposition of the “curved” (tubulin–TUB092; light gray ribbons) and “straight” (PDB ID 1JFF; light blue ribbons) tubulin conformational states. TUB092 is shown in green sphere representation. Arrows highlight regions of steric clashes between straight tubulin and TUB092.

To gain detailed insights into the binding of these chalcones to tubulin [23], we soaked compound 10 (TUB092, Figure 3A) into crystals of a protein complex composed of two αβ-tubulin (T_2_), the stathmin-like protein RB3 (R) and tubulin tyrosine ligase (TTL) [24, 25], and determined the crystal structure of the liganded TUB092 (T_2_R-TTL-TUB092) at 2.4 Å resolution (Supplementary Table 2). The overall structure of tubulin in the T_2_R-TTL-TUB092 complex superimposes with low rmsd of 0.236 Å (776 Cα atoms) onto the one obtained in the absence of the ligand (PDB-ID 4I55) [24, 25]. The binding site is formed by residues of strands S8 and S9, loop T7 and helices H7 and H8 of β-tubulin and of loop T5 of α-tubulin (Figure 3B).

The 1,3-benzodioxole ring (ring A) of TUB092 points into the hydrophobic pocket formed by the side chains of β-tubulin residues Cys241, Leu242, Leu248, Ala250, Leu255, Met259, Ala316, Ile318 Lys352, Ala354 and Ile378 and forms a water-mediated hydrogen bond to the backbone carbonyl and amide of Gly237 and Cys241, respectively (Figure 3C, 3D). The linker carbonyl of TUB092 is in hydrogen-bond distance to the backbone amide of Asp251. The hydroxyl and methoxy-groups at ring B form one direct and two water-mediated hydrogen bonds to the backbone carbonyls of Thr179 and Asn349, respectively. Moreover, both the 1,3-benzodioxole and the 2-methoxyphenol moieties are stacked between the side chains of Cys241 and Leu255, and between Asn258 and Lys352, respectively.

When the beta chains of the tubulin-colchicine and the tubulin-TUB092 structures were superimposed (Figure 3E), both the 2-methoxyphenol and the linker moieties of TUB092 overlay well with the C-ring and parts of the B and A rings of colchicine. Moreover, two methoxy- and the hydroxyl-groups of TUB092 occupy the same positions as the corresponding groups in colchicine. The simpler scaffold of TUB092 allows the T5-loop residue Thr179 of α-tubulin to flip in and occupy a space that is occupied by two water molecules in the tubulin-colchicine complex structure (Figure 3F).

### TUB091 induces apoptosis in MDA-MB-231 human breast cancer cells

**Figure 4:**
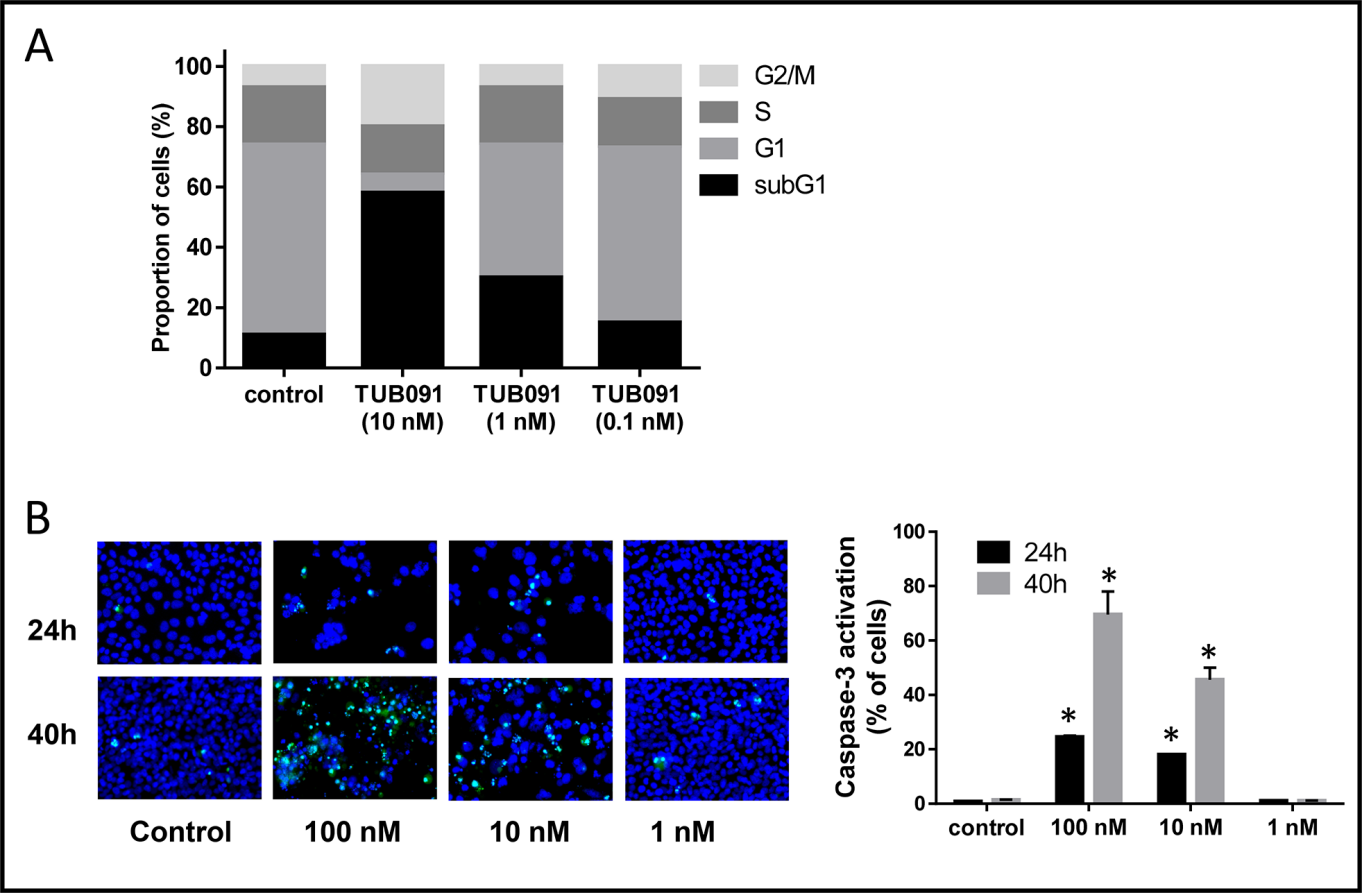
TUB091 induces G2/M phase arrest and apoptosis in human breast cancer MDA-MB-231 cells (**A**) Flow cytometric analysis of cell cycle distribution of MDA-MB-231 cells that were treated for 24 h with different concentrations of TUB091. The experiment was repeated 3 times with similar results. Results from 1 experiment are shown. (**B**) Caspase-3 activity in MDA-MB-231 cells. Different concentrations of compound and 2 μM of the caspase-3 substrate DEVD-NucView488 were added to MDA-MB-231 cells. At indicated time-points, the cells were incubated with 2 μg/ml Hoechst 33342 to stain the nucleus, and imaged. Data are the result of 3 experiments performed in duplicate and are expressed as mean ± SD. *p < 0.05 compared with control.

Tubulin staining of TUB091-treated MDA-MB-231 cell cultures (Figure 2B) suggested that the compound causes mitotic arrest. Using flow cytometry, an accumulation of cells in G2/M phase was observed after 24 h treatment with 10 nM TUB091 (Figure 4A). More strikingly, the compound caused a dose-dependent increase in sub G1 cells, exhibiting a sub-diploid DNA content characteristic of apoptotic cells (45% ± 9 and 19% ± 8 at 10 and 1 nM of TUB091, respectively). Induction of apoptosis was confirmed by measuring (i) the translocation of phosphatidylserine from the cytoplasmic to the extracellular side of the plasma membrane (Supplementary Figure 1) and (ii) the activation of caspase-3 by TUB091 (Figure 4B).

### TUB091 displays anti-vascular effects *in vitro* and in the CAM assay

**Figure 5:**
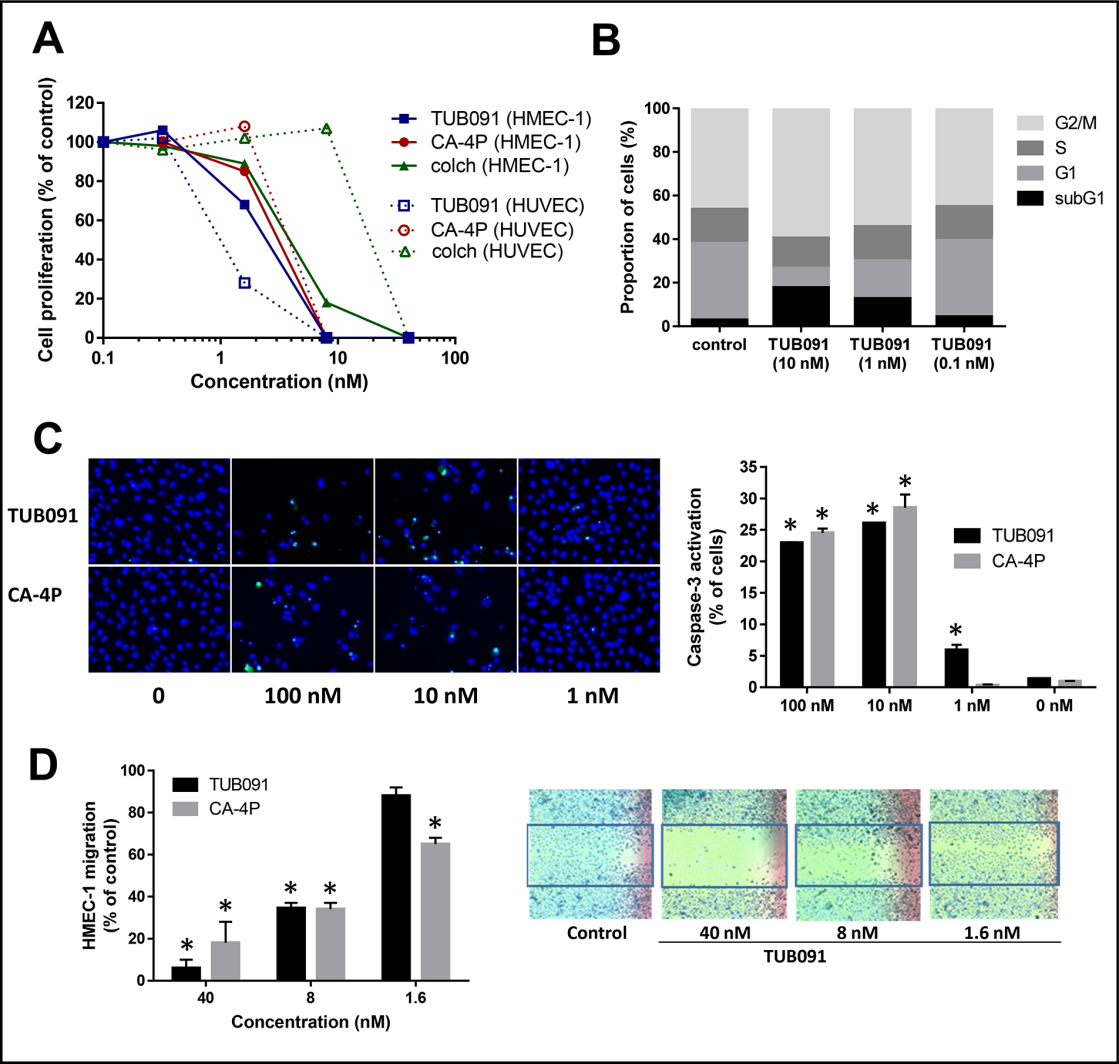
Effects of TUB091 on endothelial cell functions *in vitro* (**A**) Endothelial cell proliferation. HUVEC and HMEC-1 cells were seeded at 20,000 cells/cm^2^. After 24 h, compounds were added. The cells were allowed to grow for an additional 3 days, trypsinized and counted. Mean of 3 independent experiments with similar results is shown. (**B**) Flow cytometric analysis of cell cycle distribution of HMEC-1 cells that were treated for 24 h with different concentrations of TUB091. The experiment was repeated 3 times with similar results. Results from 1 experiment are shown. (**C**) Caspase-3 activity in HUVECs. Different concentrations of TUB091 or CA-4P and 2 μM of the caspase-3 substrate DEVD-NucView488 were added to HUVECs. At 24 h, the cells were incubated with 2 μg/ml Hoechst 33342 to stain the nucleus, and imaged. (**D**) Endothelial cell migration. Wounds were created in confluent HMEC-1 monolayers. Then, cells were incubated in fresh medium in the presence of the test compounds and 1 μg/ml of mitomycine C to inhibit cell proliferation. After 18 h, the wounds were photographed and wound repair was quantified by computerized analysis. (C, D) Data are the result of 3 experiments performed in duplicate and are expressed as mean ± SD. *p < 0.05 compared with control.

Vascular-disrupting agents not only affect tumor but also endothelial cells. Indeed TUB091, colchicine and CA-4P dose-dependently inhibited the proliferation of macrovascular HUVEC and microvascular HMEC-1 cells. Whereas the compounds showed a comparable inhibitory activity in HMEC-1 cells, TUB091 proved about 10-fold more cytostatic than CA-4P and 100-fold more active than colchicine in HUVEC cells (Figure 5A). TUB091 (≥ 10 nM) also induced the accumulation of HMEC-1 cells in G2/M phase of the cell cycle (Figure 5B) and induced apoptosis, even at 1 nM (Figure 5B, 5C). In addition, TUB091 and CA-4P prevented the closure of a wound created in a confluent monolayer of HMEC-1 cells (Figure 5D).

**Figure 6:**
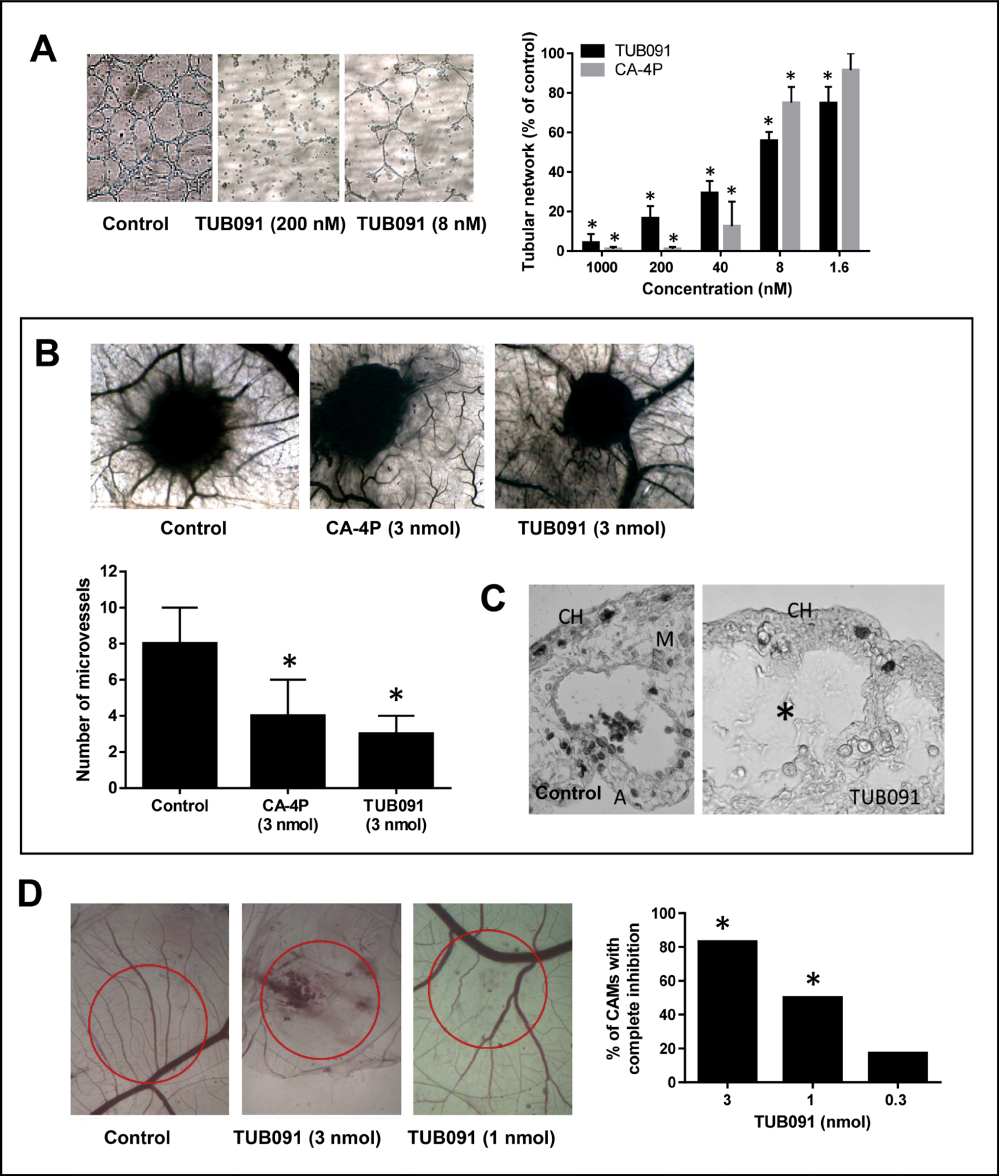
Anti-angiogenic and VDA activity of TUB091 (**A**) Disruption of the vascular network. HMEC-1 cells were cultured on matrigel for 3 h to allow the formation of tube-like structures. Then different concentrations of compounds were added. After 90 min, tube formation was quantified. Data are the result of 3 experiments performed in duplicate and are expressed as mean ± SD. *p < 0.05 compared with control. (**B**–**C**) Gelatin sponge CAM assay. Sponges containing 3 nmol of CA-4P or TUB091 were added onto the CAM. (B) Newly formed microvessels directed at the sponges were counted 4 days later (lower panel). Hundred eggs were used per condition. Data are expressed as mean ± SD. *p < 0.05 compared with control. Macroscopic pictures of the CAM are shown (upper panel) (C) Histological examination demonstrates a complete disappearance of the intermediate mesenchyme without any cellular and vascular components in the TUB091-treated CAMs, as compared to control. CH: chorion; M: mesenchyme, A: allantois. (**D**) Plastic discs containing the dried compound were added onto the CAM. Graph shows the percentage of CAMs with complete inhibition of blood vessel formation 2 days later. Data are the result of 2 independent experiments using 8 eggs per condition. *p < 0.05 compared with control.

As shown in Figure 6A, both CA-4P and TUB091 dose-dependently abrogated the integrity of a pre-formed vascular network of endothelial cells. TUB091 also elicited anti-angiogenic and vascular-disrupting effects *in vivo*. Using the CAM gelatin sponge assay, the number of newly formed microvessels directed towards the sponge containing 3 nmol of TUB091 or CA-4P was reduced by more than 50% compared with DMSO-containing sponges (Figure 6B). Histological examination of the CAMs showed a complete disappearance of the intermediate mesenchyme without any cellular and vascular components in the TUB091-treated CAMs, as compared to control (Figure 6C). Using larger plastic discs that contain the dried compound, both neovessel formation (angiogenesis) as well as vascular-disrupting activity can be investigated at the CAM area under the disc. A dose-dependent effect on both processes could be observed. Indeed, at 3 nmol, TUB091 completely blocked the formation of new blood vessels in nearly all CAMs tested and also caused damage and bleeding to immature, already existing vessels (Figure 6D). At 1 nmol/ disc, angiogenesis was inhibited, but larger vessels were not affected (Figure 6D).

### Prodrug synthesis

All these data point to a vascular-disrupting and anti-angiogenic effect of TUB091 accompanied by induction of tumor cell apoptosis. However, its low aqueous solubility precluded *in vivo* evaluation. Therefore, we synthesized prodrugs of TUB091 by conjugation with L-Ser as in AVE-8062 (3) or with an L-Lys-L-Pro dipeptide (12 and 13, Supplementary Methods). The latter was based on our previous results of solubility improvement of antiviral drugs by coupling to di- or oligopeptides and subsequent release by the endogenous enzyme dipeptidyl peptidase IV (DPP-IV/CD26) [26].

**Figure 7:**
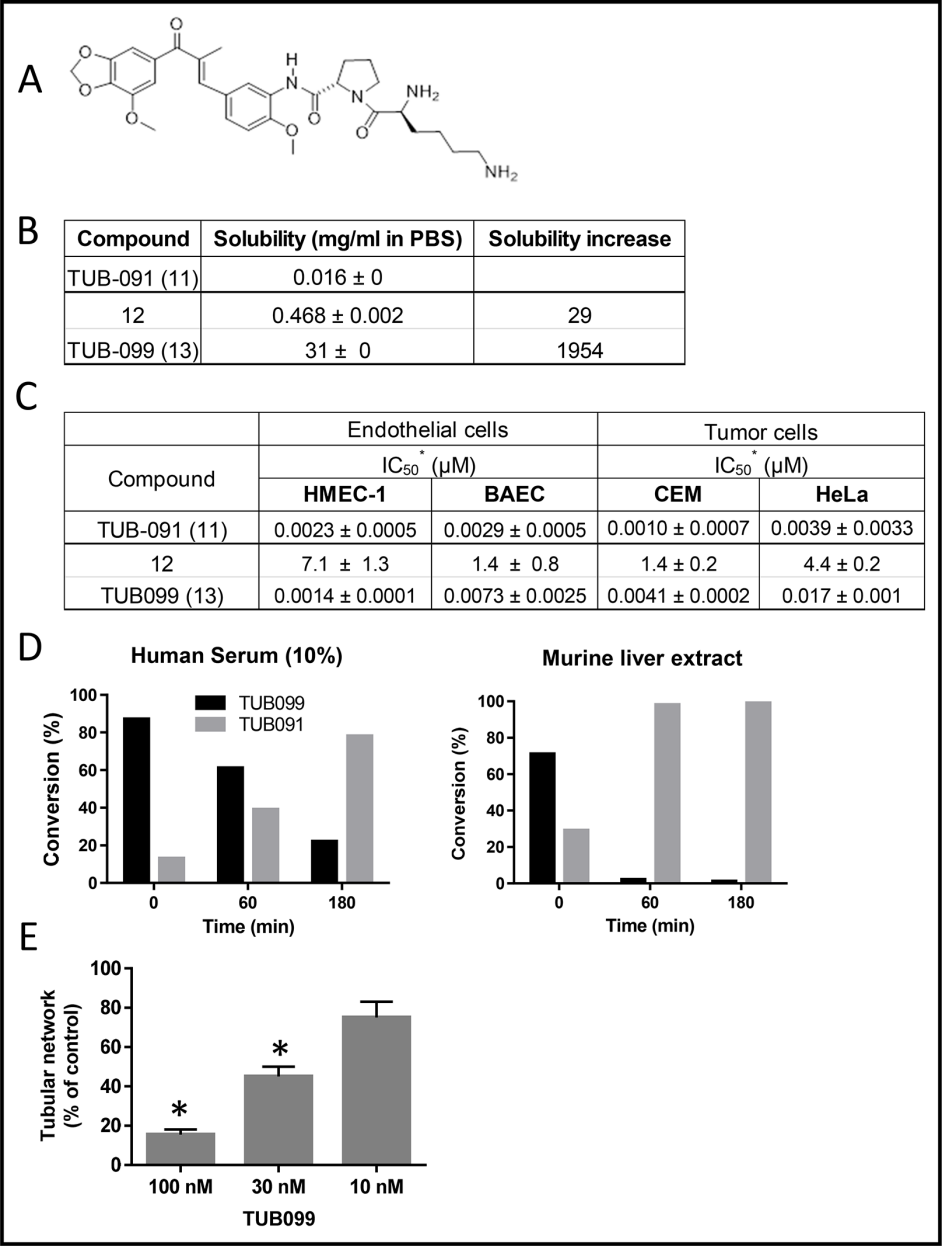
Biological evaluation of TUB091 prodrugs (**A**) Structure of TUB099, the L-lysyl-L-prolyl derivative of TUB091. (**B**) Aqueous solubility of TUB091 and its prodrugs in PBS. (**C**) Growth-inhibitory activity is presented as IC_50_, i.e. concentration that reduces cell growth by 50%. HMEC-1: human microvascular endothelial cell line-1; BAEC: bovine aortic endothelial cells; Cem: human lymphocytic leukemia cells; HeLa, human cervical carcinoma cells. Data are mean ± SD. (**D**) Release of parent compound (TUB091) in human serum and murine liver extract. TUB099 (100 μM) was incubated for 60 and 180 min at 37°C in human serum or mouse liver extract. Aliquots were quantified by HPLC with detection at 305 nm. (**E)** Vascular-disrupting activity of TUB099. HMEC-1 cells were cultured on matrigel for 3 h to allow the formation of tube-like structures. Then different concentrations of TUB099 were added. After 90 min, tube formation was quantified. Data are the result of 3 experiments performed in duplicate and are expressed as mean ± SD. *p < 0.05 compared with control.

The L-Lys-L-Pro derivative 13 (TUB099, Figure 7A) was ~2000-fold more soluble than the parent drug (Figure 7B) and nearly as effective as TUB091 in inhibiting tumor and endothelial cell proliferation (Figure 7C). Incubation of TUB099 in human serum or murine liver extract showed an efficient release of TUB091 (Figure 7D). Biological assays further demonstrated that TUB099 possesses vascular-disrupting activity *in vitro* (Figure 7E) and induces G2/M phase arrest and apoptosis in endothelial and tumor cells (not shown).

### TUB099 inhibits primary tumor growth and spontaneous metastasis in mice

**Figure 8:**
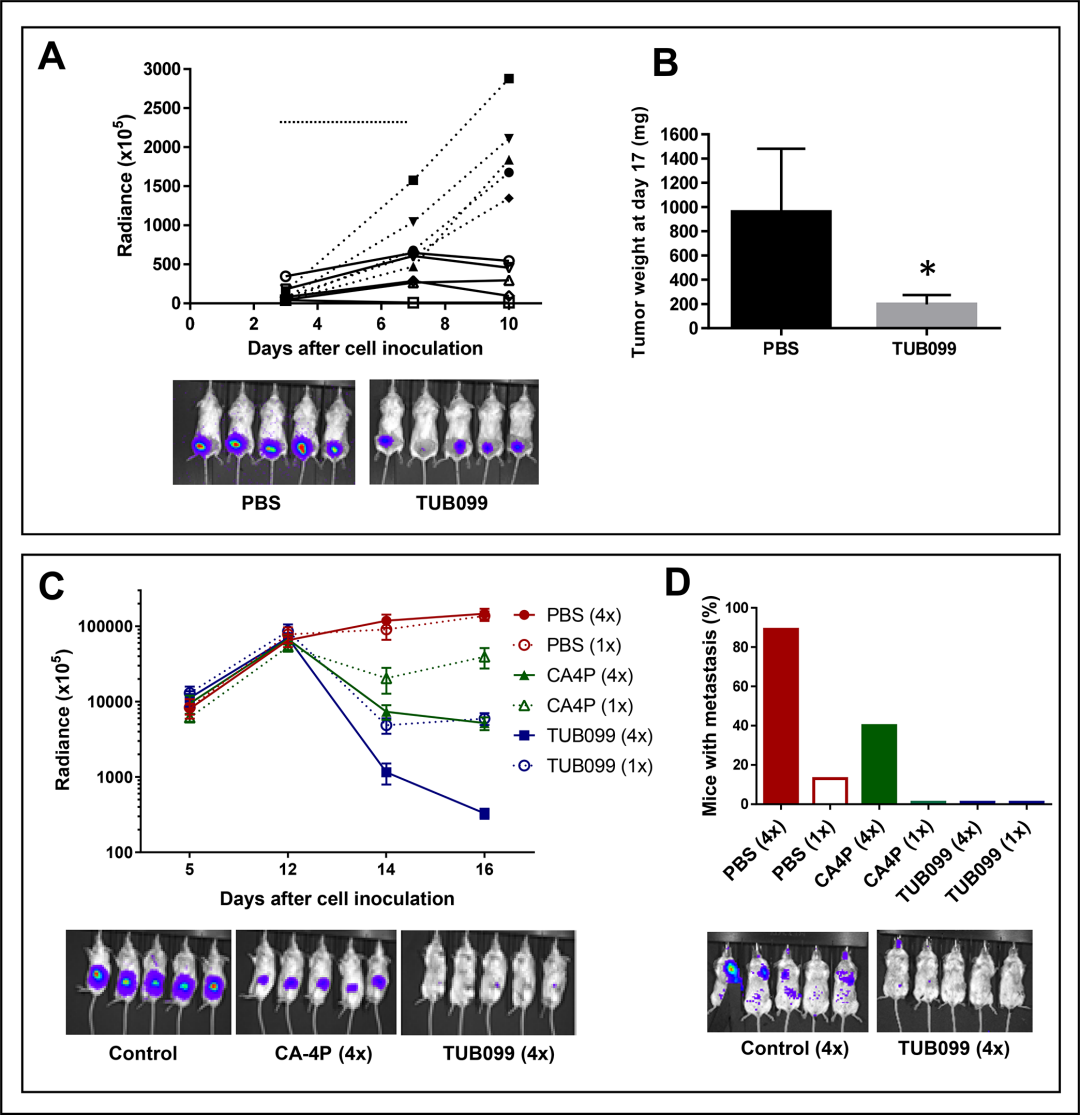
Intratumoral (i.t.) TUB099 impairs primary tumor growth and metastasis (**A**, **B**) Effect of TUB099 on subcutaneous growth of melanoma. Thirty thousand B16.F10.luc2 cells were injected subcutaneously in SCID mice. Intratumoral (i.t.) treatment with TUB099 (10 mg/kg; full line) or vehicle (dashed line) was started 3 days after cell injection and continued for 5 consecutive days (horizontal line in A). The mice were imaged (A) at regular time intervals. Each line in A represents one single mouse. Representative pictures of bioluminescence in the tumors of control and TUB099-treated mice at day 10 are shown. (B) Tumor weight at day 17 after cell inoculation, i.e. 10 days after treatment was terminated. Data are mean ± SEM, *n* = 5. *p < 0.05. (**C**, **D**) Effect of TUB099 on primary tumor growth and metastasis of MDA-MB-231/4mRL.luc2 human breast cancer cells. 4mRL.luc2 cells (10^6^) were injected in the mammary fat pad of SCID mice. PBS, TUB099 or CA-4P was administered i.t. at day 12 only (1×) or from day 12 till day 15 (4×). (C) The mice were imaged at regular time intervals. Data are mean ± SEM of luciferase measurements, *n* = 5. At day 16, a significant reduction in luminescent signal is visible in the CA-4P- but particularly in the TUB099-treated tumors. (D) Lung and/or lymph node metastasis 19 days after removal of the primary tumor (i.e. 35 days after cell inoculation). Experiments were performed twice with similar results. Results of one experiment are shown.

To determine the *in vivo* antitumor activity of TUB099, melanoma and breast cancer models were used [27]. First, B16-F10.luc2 melanoma cells were injected subcutaneously (s.c.) in SCID mice. TUB099 was injected intratumorally (i.t.) at 10 mg/kg for 5 consecutive days starting at day 3, when small tumors were macroscopically visible. Tumor growth was measured by *in vivo* imaging until day 10, when TUB099-treated tumors were no longer visible (Figure 8A). By day 17, TUB099 tumors had slightly regrown but were significantly smaller than control tumors (*p* < 0.001, Figure 8B). Only a slight, non-significant, decrease in body weight was noted (Supplementary Figure 2). Thus, in an early stage of tumor development, TUB099 exerts a potent anti-tumor effect when administered i.t.

Next, MDA-MB-231/4mRL.luc2 human breast cancer cells were injected into the mammary fad pad of SCID mice and the compounds (TUB099 or CA-4P) were administered i.t. at 15 mg/kg either once at day 12, or for 4 consecutive days (day 12 till 15). Whereas control tumors continued to grow, a dramatic decrease in luminescent signal was noted in the TUB099 group within 2 days after start of treatment (Figure 8C). TUB099 proved also significantly more active than the reference compound CA-4P. However, caliper measurement of tumor size did not reveal a difference between control and treated groups (not shown). Since light emission by firefly luciferase requires oxygen and ATP, which are only present in living cells, our data indicate that, already within 48 h after start of treatment, TUB099-treated tumor cells were dead and/or lacked oxygen, effects that are typically seen with VDAs.

At day 16 after cell inoculation, primary tumors were removed and all mice were left untreated. Three weeks later, lung and/or lymph node metastases were present in 90% of control mice of the 4-day treatment group. In contrast, only 40% of CA-4P-treated mice developed metastases, and metastases were completely undetectable (based on luminescence measurement) in TUB099-treated mice (Figure 8D). Interestingly, among the 1-day treatment groups, metastases development was less evident.

### TUB099 causes rapid vascular shutdown in MDA-MB-231 orthotopic breast tumors

**Figure 9:**
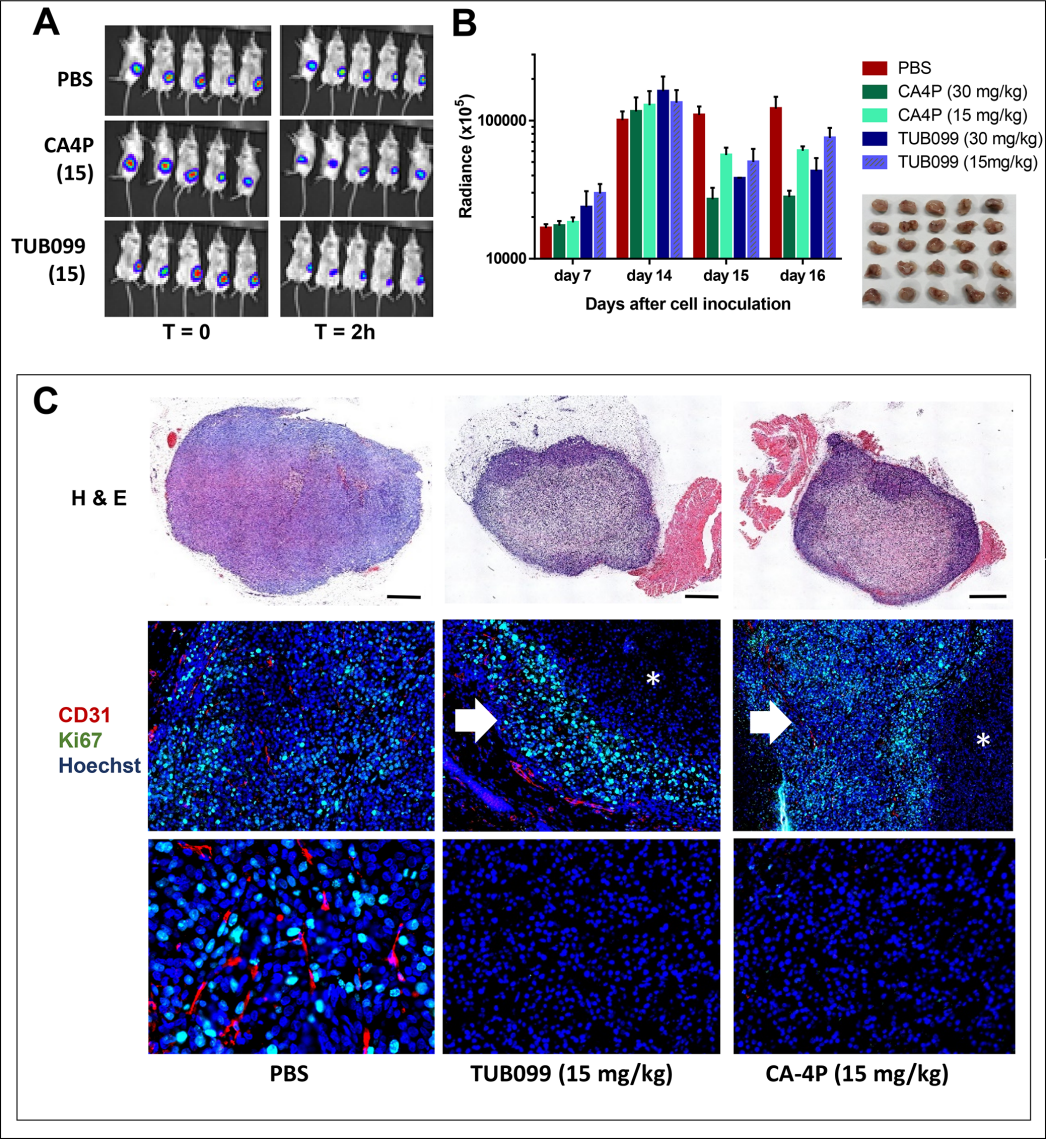
Vascular disrupting activity of systemic (i.p.) TUB099 treatment (**A**–**C**) MDA-MB-231/4mRL.luc2 cells (10^6^) were injected in the mammary fat pad of SCID mice. PBS, TUB099 or CA-4P (15 or 30 mg/kg) was administered once, intraperitoneally, at day 14. (A) Luminescence in the tumor before (left) and 2 h after administration (right) of compound. (B) The mice were imaged at regular time intervals. Data are mean ± SEM, *n* = 5.No difference in primary tumor size was observed at day 16, when the tumors were resected and processed for histological evaluation (inset). (C) Total tumor sections were stained with H&E or were double immunostained with anti-CD31 (red) and anti-Ki67 (green) antibodies, followed by nuclear counterstaining with Hoechst (blue). Asterisks indicate necrotic tumor center. Higher magnification of tumor center (bottom panel). Arrow indicates viable tumor rim. Scale bar = 1 mm. Two independent experiments yielded comparable data.

Next, mice were treated systemically (i.p.) with one dose (30 or 15 mg/kg) of TUB099 or CA-4P on day 14 after cell injection. A massive decline in luminescent signal was noted as soon as 2 h after treatment (Figure 9A), in agreement with the rapid vascular shutdown and consequently reduced perfusion induced by VDAs. This effect was still evident 24 and 48 h after treatment (i.e. day 15 and 16 respectively), in particular in the 30 mg/kg groups (Figure 9B). Again, when the primary tumors were removed and processed for histology, no difference was observed in tumor size at day 16 (Figure 9B).

H&E staining indicated necrosis in the center of TUB099- and CA-4P-treated tumors, leaving a viable rim, whereas control tumors were entirely viable. In addition, while control tumors were highly vascularized and showed massive staining of the proliferation marker Ki67 (Figure 9C), tumors obtained from CA-4P and TUB099-treated mice showed very little staining for Ki67, and no blood vessels in the tumor center (Figure 9C).

Again primary tumors were removed at day 16. By day 21 after tumor resection, 45% of the control mice were metastasized. CA-4P showed an anti-metastatic activity at 30 mg/kg (13% of mice with metastasis), whereas TUB099 proved equally effective in inhibiting metastasis at 15 and 30 mg/kg (20% of mice with metastasis) (not shown).

Together, our data indicate that TUB099 induces rapid intratumoral vascular collapse, leading to massive tumor necrosis, with a potency similar to that of the reference compound CA-4P. Moreover, TUB099 effectively reduced spontaneous metastasis, occurring after resection of the primary tumors.

## DISCUSSION

The growth of solid tumors and their metastatic dissemination are dependent on a functional blood supply. Therefore, the tumor vasculature is considered an attractive target for therapy [3, 28]. During the past decades much effort has been devoted to the development and clinical evaluation of anti-angiogenic drugs, which inhibit the formation of new blood vessels [28, 29]. However, these compounds have largely failed in the clinic because of resistance development, lack of efficacy and/or toxicity. A complimentary approach also targeting the tumor vasculature is the use of VDAs. These agents destroy the existing, abnormal intratumoral vasculature, leading to rapid vascular shutdown and extensive tumor necrosis [3, 5]. The largest group of VDAs contains microtubule-targeting agents, of which CA-4P is currently under investigation in phase II clinical trials for a variety of solid tumors [5, 30].

Here, we investigated the tubulin-binding, vascular-targeting, anti-tumor and anti-metastatic activities of a new series of chalcone derivatives. The best compound of this series, TUB091, inhibited the proliferation of endothelial and tumor cell lines with 50% inhibitory concentrations of 1–4 nM, which is slightly better than the reference compounds colchicine and CA-4P.

Using X-ray crystallography we showed that the chalcones bind to tubulin at the colchicine-site. While free tubulin dimers adopt a characteristic “curved” conformation in solution [31, 32], tubulin dimers that are incorporated in microtubules undergo a curved-to-straight structural transition [33, 34]. This process is necessary for microtubule formation and is associated with a rearrangement of several secondary structural elements thereby occluding the colchicine-site [23, 35, 36]. The superposition of the modeled tubulin-TUB092 structure in the context of the straight conformational state of tubulin showed several potential clashes of TUB092 with residues from the helices H8 and H7, the beta strand S9 and the T7 loop of β-tubulin and from the T5 loop of α-tubulin. This observation suggests that TUB092 cannot bind to the colchicine-site in preformed microtubules. Thus, the microtubule-destabilizing activity of TUB092 likely consists in preventing the curved to-straight structural transition in tubulin by the bound ligand, as previously reported for other colchicine-site ligands [23, 36].

Together, our data demonstrate that the newly synthesized chalcones destabilize microtubules by binding to the colchicine-site of tubulin. This destabilization causes depolymerization of the mitotic spindles leading to the lack of DNA organization into a metaphase plate. Immunocytochemistry showed a dose-dependent increase in (pro)metaphase cells containing abnormal mitotic spindles, in the presence of TUB091. Accordingly, TUB091 caused an arrest of tumor and endothelial cells in G2/M phase and induced apoptosis in these cells at nanomolar concentrations.

Furthermore, we found a potent and dose-dependent inhibition of other endothelial cell-specific responses. Indeed, TUB091 inhibited endothelial cell migration and angiogenesis in the CAM assay. The compound also caused the disruption of an existing network of endothelial tubes *in vitro* and the degradation of newly formed, immature vessels in the CAM assay. In contrast, the larger, mature vasculature was not affected by TUB091. Indeed, significant structural differences exist between immature and mature vasculature. While immature vessels rely on microtubules for structural support and are particularly sensitive to VDAs, mature vessels are supported by pericytes and a solid basement membrane and are therefore protected from disruption by VDAs [37].

Previously, prodrugs of combretastatins were developed to overcome solubility limitations that precluded clinical testing. Sodium phosphate salt derivatives of CA-4 (CA-4P) and CA-1 (CA-1P) and the L-serine prodrug of an amino derivative of CA-4 (AVE-8062, rapidly advanced to clinical trials [reviewed in [5]). We have extensively investigated the development of oligopeptide prodrugs that are efficiently release in the presence of dipeptidyl peptidase IV (DPP-IV/CD26) [26]. This enzyme, abundantly found in serum, mainly hydrolyzes peptidic sequences containing an *N*-terminal penultimate proline. In particular, drug conjugation with an L-lysyl-L-prolyl dipeptide may significantly increase the solubility due to the basicity of the *N*-terminal lysine, while recognition by CD26 may enhance the *in vivo* release of the parent drug. Indeed, the L-lysyl-L-prolyl dipeptide prodrug of TUB091 (TUB099) displayed 2000-fold improved solubility compared to TUB091 and efficiently released the parent drug. This prodrug inhibited endothelial and tumor cell proliferation, induced apoptosis and showed vascular-disrupting activity in cell culture.

In addition, when administered either intratumorally (i.t.) or systemically (i.p.), TUB099 reduced the primary growth of mouse melanoma B16.F10.luc2 and human breast cancer MDA-MB-231/4mRL.luc2 cells in mice. Whereas i.t. treatment of small tumors with TUB099 resulted in complete regression within 4 days, no immediate macroscopic effect on tumor size was noted when larger tumors were treated with TUB099 or CA-4P. Interestingly, already 2 hours after compound injection, a prominent reduction in luminescence was observed, indicative of vascular shutdown and consequently reduced tumor cell perfusion. Accordingly, whereas PBS-treated tumors were highly proliferative and vascularized, as demonstrated by extensive Ki67 and CD31 staining, immunohistochemical analysis of tumors dissected 2 days after treatment with TUB099 and CA-4P showed massive necrosis in the tumor center, leaving only a small viable rim. These effects are typically seen with vascular-targeting agents [37]. Since these peripheral tumor cells are more accessible to conventional chemotherapy, combretastatins are mainly used to complement traditional anticancer treatments, including various cytostatic agents or radiotherapy (reviewed in [5]).

To evaluate the effect of TUB099 on spontaneous metastasis, primary tumors were removed after treatment. Whereas some PBS-treated tumors regrew during the next weeks, the mice that were previously treated with either TUB099 or CA-4P remained tumor-free. Furthermore, 87% of control mice developed lung and/or lymph node metastases within 4 weeks after tumor resection, whereas metastases were only visible in 40% of CA-4P treated animals and were completely absent in the TUB099 group. Interestingly, much less metastases were observed in the control mice receiving just one i.t. injection of PBS, compared to the mice that received PBS for 4 consecutive days. These data suggest that i.t. injection may damage tumor architecture and the intratumoral vasculature, thereby facilitating the escape of tumor cells into the circulation and their dissemination to distant organs [39].

Taken together, our data indicate that this series of chalcones open a new horizon for the design of innovative VDAs, since their interaction with their molecular target has also been resolved and presented. Interestingly, they show a superior antitumor activity over the reference VDA CA-4P. Finally, we provide evidence that our prodrug approach, that so far has been mainly used to develop more soluble antivirals, may also be very valuable to improve bioavailability of highly hydrophobic anti-cancer drugs.

## MATERIALS AND METHODS

### Compounds

Colchicine was obtained from Calbiochem (Darmstadt, Germany) and CA-4P (fosbretulin) from Sigma-Aldrich (Diegem, Belgium). The synthesis, analytical and spectroscopic data of compounds 5–13 are shown in the Supplemental Experimental Procedures.

### Cell lines

Human umbilical vein endothelial cells (HUVEC) were purchased from Lonza and grown in macrovascular endothelial cell medium (EGM2, Lonza, Verviers, Belgium). The human microvascular endothelial cell line HMEC-1 was obtained at passage 12 from the Centers for Disease Control and Prevention (Atlanta, GA, USA) and used from passage 17 till 27. Bovine aortic endothelial cells (BAEC) were purchased from Lonza at passage 3 and were used up to passage 20. Human embryonic lung HEL-299 fibroblasts were obtained from ATCC, used up to passage 10 and cultured in Dulbecco's modified Eagle's medium (DMEM; Gibco, Carlsbad, CA, USA), supplemented with 10% fetal bovine serum (FBS, Gibco), 0.01M Hepes (Gibco) and 1 mM sodium pyruvate (Gibco). Buffy coat preparations from healthy donors were obtained from the Blood Transfusion Center in Mechelen, Belgium. Peripheral blood mononuclear cells (PBMC) were isolated by density gradient centrifugation over Lymphoprep (*d* = 1.077 g/ml) (Nycomed, Oslo, Norway) and cultured in RPMI 1640 containing 10% FBS and 2 mM L-glutamine. Luciferase-expressing murine melanoma (B16-F10.luc2) and human breast carcinoma (MDA-MB-231.luc2) cells were obtained from Perkin Elmer (Zaventem, Belgium). A metastatic MDA-MB-231.luc2 cell line (231/4mRL.luc2) was derived by serial *in vivo* selection of metastases that emerged following resection of the primary orthotopic tumor according to Munoz et al. [40]. Human cervical carcinoma (HeLa) and human T-lymphoid (CEM) cells were obtained from ATCC (Middlesex, UK). Cell lines were maintained in culture for up to 3 months and grown in DMEM, supplemented with 10% FBS, 0.01M Hepes and 1 mM sodium pyruvate. All cells were cultured in a humidified 5% CO_2_ incubator at 37°C. No authentication was done since the cell lines were purchased.

### Cell growth inhibition and cytotoxicity studies

BAEC, MDA-MB-231, B16.F10.luc2 and HeLa cells were seeded at 10,000 cells/cm^2^. HUVEC, HMEC-1 and Hel cells were seeded at 20,000 cells/cm^2^. After 24 h, 5-fold dilutions of the compounds were added. Three days (4 days for HUVEC and HMEC-1 and 6 days for Hel) later the cells were counted by means of a Coulter counter (Analis, Belgium). CEM cells were seeded at 180,000 cells/cm^2^ in the presence of the compounds, allowed to proliferate for 96 h and counted. One day after isolation, PBMC were stimulated with phytohemagglutinin (2 μg/ml) for 48 h. Next, cells were seeded at 450,000 cells/cm^2^, treated with compound and interleukin-2 (20U/ml) for 6 days and counted.

Cytotoxicity was studied using the xCELLigence RTCA DP instrument (Acea Biosciences, San Diego, CA, USA). 100 μl of cell-free growth medium was added to the wells of an E-plate VIEW 16 PET (Acea Biosciences) and allowed to equilibrate for 1 h for background impedance measurement. Next, 4,000 MDA-MB-231 cells/100 μl were added to the wells. After overnight incubation, 50 μl of compound was added. The impedance value was monitored every 15 min for 24 h and expressed as Cell index (CI). CI was normalized at the time of addition of the compound.

### Immunocytochemistry

MDA-MB-231 cells were seeded overnight on poly-L-Lysine coated 8-well Millicell slides (Millipore). Next, compounds were added for 15 h. Fixed and permeabilized cells were stained with a monoclonal anti-β-tubulin antibody (2 μg/ml, Sigma-Aldrich) for 2 h at RT. After washing, cells were incubated with goat anti-mouse Alexa Fluor 488 antibody (4 μg/ml; Molecular Probes, Invitrogen) as described [22]. Nuclei were stained with 2 μg/ml Hoechst33342 (Sigma-Aldrich). Fluorescence microscopy was done using an Axiovert 200 M inverted microscope (Zeiss, Göttingen, Germany).

### Tubulin polymerization-determination of binding constants

Calf brain tubulin was purified as described [22, 41]. (R)-(+)-ethyl 5-amino 2-methyl-1, 2-dihydro-3-phenylpyrido[3, 4-b]pyrazin-7-yl carbamate (R-PT) was a kind gift of Prof. Rener, Birmingham, Alabama [42].

The binding constant of TUB092 to tubulin was determined by competition with R-PT [21, 22]. The binding constant of TUB091 to tubulin was determined by competition with TUB092. The fluorescence emission spectra (excitation at 374 nm) of 0.4 μM Tubulin and 0.4 μM TUB091, 0.1 mM GTP pH 7.0, were measured in the presence of growing concentrations of TUB092 using a Jobin-Ybon SPEX Fluoromax-2 (HORIBA, Ltd. Kyoto, Japan). The decrease of the fluorescence intensity of TUB091 at the maximum upon incubation with TUB092 was used to determine the binding constant using Equigra V5.0 as described [43].

### Crystallization, data collection, and structure solution

Crystals of T_2_R-TTL were generated as described [24, 25, 36]. Suitable T2R-TTL crystals were exchanged into reservoir solutions containing 2 mM TUB092 and soaked overnight. Soaked crystals were flash cooled in liquid nitrogen following a brief transfer into cryo solution containing 20% glycerol. T_2_R-TTL TUB-92 data were collected at beamline X06SA at the Swiss Light Source (Paul Scherrer Institut, Villigen, Switzerland). Images were indexed and processed using XDS [44]. Structure solution using the difference Fourier method and refinement were performed using PHENIX [45]. Model building was carried out iteratively using the Coot software [46]. Data collection and refinement statistics are given in Supplementary Table 1. Coordinates of the T2R-TTL-TUB092 complex have been deposited at the Protein Data Bank (PDB) under accession number 5JVD.

### Structural analysis and figure preparation

Molecular graphics and analyses were performed with PyMol (The PyMOL Molecular Graphics System, Version 1.5.0.5. Schrödinger, LLC). Chains in the T_2_R-TTL complex were defined as follows: chain A, α-tubulin-1; chain B, β-tubulin-1; chain C, α-tubulin-2; chain D, β-tubulin-2; chain E, RB3; chain F, TTL.

### Cell cycle analysis

MDA-MB-231 or HMEC-1 cells were seeded overnight at 15,000 cells/cm^2^ or 20,000 cells/cm^2^, respectively. Next, compounds were added for 24 h. The cells were stained with propidium iodide using the CycleTEST PLUS DNA Reagent Kit (BD Biosciences, San Jose, CA) [22] and their DNA content assessed using a FACSCalibur flow cytometer (BD Biosciences).

### Fluorescence detection of caspase-3 activity in live cells

MDA-MB-231 cells or HUVECs were seeded at 40,000 cells/cm^2^ or 15,000 cells/cm^2^ respectively. After 24 h, cells were incubated in Fluorobrite (Gibco)-containing growth medium or HUVEC growth medium, with the compound and 2 μM of the caspase-3 substrate DEVD-NucView488 (Biotium, Hayward, CA). At indicated time-points the DNA was stained with Hoechst33342 and the cells were imaged by fluorescence microscopy.

### Wound healing (scratch assay)

Wounds were created in confluent HMEC-1 monolayers with a 1.0-mm wide micropipette tip. Then, cells were incubated in fresh medium containing the compounds and 1 μg/ml of mitomycine C. After 18 h, wounds were photographed and wound repair was quantified by computerized analysis.

### Tube destruction

Wells of a 96-well plate were coated with 70 μl matrigel (10 mg/ml, BD Biosciences, Heidelberg, Germany) at 4°C. After gelatinization at 37°C during 30 min, HMEC-1 cells were seeded at 60,000 cells/well on top of the matrigel. After 3 h of incubation at 37°C, when tube-like structures were detectable, compounds were added. Ninety minutes later, tube destruction was evaluated by giving a score from 0 to 3 (3: intact tubular network; 2: missing connections and/or dead ends; 1: many separate, small tubes that are not connected; 0: no tubes) [22].

### Chorioallantoic membrane (CAM) assay

The *in vivo* CAM angiogenesis model was performed as described [47, 48]. At day 9 of fertilization, sterile plastic discs containing either vehicle or the compound and cortisone acetate (100 μg/disc, Sigma, St. Louis, MO) were placed on the CAM. Next, the eggs were incubated until day 11 when the area around the discs was cut-off and photographed. Results were analyzed by two-tailed paired Student's *t*-test. In another series of experiments, gelatin sponges were used as vehicle, and the number of blood vessels converging towards the sponge were quantified, as described previously (48). For histological examination, the CAM was fixed in ovo in Bouin's fluid, removed and processed for embedding in paraffin. Ten-micrometer serial sections were cut parallel to the surface of the CAM and observed under light microscope without staining.

### Solubility of the compounds

An excess amount of the tested compound was added to 400 μL PBS (1% DMSO), shaken at RT for 2 h and centrifuged. An aliquot of each supernatant was analyzed by UV (Thermo Multiskan Spectrum) by comparison with a five-point standard calibration curve.

### Stability of the compounds

The test compounds (100 μM) were incubated for 60 and 180 min at 37°C in concentrated mouse liver extract (50 % in PBS in a final volume of 200 μl) or human serum (10 % in PBS in a final volume of 300 μl). Next, 50 μl of the reaction mixture were withdrawn and added to 100 μl ice-cold methanol for another 10 min. Then, the mixtures were centrifuged for 10 min at 10,000 rpm, and the supernatants evaporated in a Speedvac apparatus (Savant; Werchter, Belgium). The dried residue was resolubilized in PBS and analysed on a C18 reverse phase column (LiChroCard, Merck, Darmstadt, Germany) by HPLC (Waters, Milford, MA) using a gradient system of water/acetonitrille (50%). Compound detection was performed at 305 nm.

### Animals

Female severe combined immunodeficient (SCID) mice, weighing about 20 g were used. The animals were bred at the animal facility of the Rega Institute. All studies were done in compliance with the ethical guidelines for animal welfare of the KU Leuven (P028/2011).

### Primary tumor models

Thirty thousand B16-F10-luc2 cells were injected subcutaneously (s.c.) in SCID mice. Intratumoral (i.t.) treatment with vehicle or TUB099 (10 mg/kg) was started 3 days after cell injection and continued for 5 consecutive days.

MDA.MB.231/4mRL.luc2 cells (10^6^) were suspended in 50 μL of 50% matrigel/PBS and injected in the mammary fat pad of SCID mice. The mice were treated intraperitoneally (i.p.) with vehicle, TUB099 or CA-4P at day 14 or treated i.t. at day 12 or at day 12 till day 15. At day 16, primary tumors were removed and processed for histological examination. The growth of the luciferase-positive tumor cells and the appearance of metastases were quantified using the IVIS spectrum imaging system (Caliper Life Sciences, Hopkinton, MA, USA) [27, 49]. Before imaging, the mice were injected s.c. with 200 μl of luciferin (15 mg/ml in PBS).

### Immunohistochemical analysis of the tumors

IHC staining of the tumors was performed as described [27]. Paraffin-embedded sections were stained ON at 4°C with a rabbit anti-CD31 (4 μg/ml, Abcam) and a rat anti-Ki67 (1 μg/ml, eBioscience) antibody. After washing, sections were incubated for 3 h at RT with Alexa Fluor 568 goat anti-rabbit antibody (4 μg/ml; Molecular probes) and Dylight 650 conjugated goat anti-rat antibody (4 μg/ml; Thermofisher) and examined by fluorescence microscopy.

## SUPPLEMENTARY MATERIALS FIGURES AND TABLES



## Abbreviations

BAEC: bovine aortic endothelial cell
CA-4: combretastatin A4
CAM: chicken chorioallantoic membrane
DPP-IV/CD26: dipeptidyl peptidase IV
HUVEC: human umbilical vein endothelial cell
HMEC-1: human microvascular endothelial cell line
IC_50_: 50% inhibitory concentration
i.p.: intraperitoneal
i.t.: intratumoral
R: stathmin-like protein RB3
s.c.: subcutaneous
T_2_: αβ-tubulin
TTL: tubulin tyrosine ligase
VDAs: vascular-disrupting agents
