# Supplementary material for: Antivascular and antitumor properties of the tubulin-binding chalcone TUB091
Source: Oncotarget. 2016 May 20;8(9):14325–42. doi: 10.18632/oncotarget.9527 (PMC5362409; doi:10.18632/oncotarget.9527)
Supplement: Supplementary file 1 [file oncotarget-08-14325-s001.pdf]

Antivascular and antitumor properties of the tubulin-binding chalcone TUB091

Supplementary Materials

SUPPLEMENTARY EXPERIMENTAL PROCEDURES

Cells

Human embryonic lung HEL-299 fibroblasts were obtained from ATCC, used up to passage 10 and cultured in Dulbecco's modified Eagle's medium (DMEM; Gibco, Carlsbad, CA, USA), supplemented with 10% fetal bovine serum (FBS, Gibso), 0.01M Hepes (Gibco) and 1 mM sodium pyruvate (Gibco). Buffy coat preparations from healthy donors were obtained from the Blood Transfusion Center in Mechelen, Belgium. Peripheral blood mononuclear cells (PBMC) were isolated by density gradient centrifugation over Lymphoprep (*d* = 1.077 g/ml) (Nycomed, Oslo, Norway) and cultured in RPMI 1640 containing 10% FBS and 2 mM L-glutamine

Cell growth inhibition

Hel cells were seeded at 20,000 cells/cm^2. After 24 h, 5-fold dilutions of the compounds were added. Six days later the cells were counted by means of a Coulter counter (Analis, Belgium). One day after isolation, PBMC were stimulated with phytohemagglutinin (2 µg/ml) for 48 h. Next, cells were seeded at 450,000 cells/cm^2, treated with compound and interleukin-2 (20 U/ml) for 6 days and counted.

Annexin V-FITC/propidium iodide staining

To differentiate between normal (living), apoptotic and necrotic cells, exponentially growing MDA-MB-231 cells (seeded at 20,000 cells/cm^2) were incubated for 24h in the presence of different concentrations of compound. Cells were then simultaneously stained with Annexin-V-FITC and PI using the Annexin-V-FITC staining kit (Sigma), and analyzed by flow cytometry as described previously [1]. Early apoptotic cells are characterized by high Annexin binding and low PI staining (lower right quadrant), whereas late apoptotic (upper right quadrant) and necrotic cells (upper left quadrant) stain strongly for both Annexin and PI.

Animal model

Thirty thousand B16-F10-luc2 cells were injected subcutaneously in SCID mice. Intratumoral (i.t.) treatment with TUB099 (10 mg/kg) was started 3 days after cell injection and continued for 5 consecutive days. The same volume of phosphate-buffered saline (PBS) was given to control mice. Body weight was determined twice weekly.

Crystallization, data collection, and structure solution

Crystals of T_2R-TTL were generated as described [2–4]. Suitable T_2R-TTL crystals were exchanged into reservoir solutions containing 2 mM TUB092 and soaked overnight. Soaked crystals were flash cooled in liquid nitrogen following a brief transfer into cryo solution containing 20% glycerol. T_2R-TTL TUB092 data were collected at beamline X06SA at the Swiss Light Source (Paul Scherrer Institut, Villigen, Switzerland). Images were indexed and processed using XDS [5]. Structure solution using the difference Fourier method and refinement were performed using PHENIX [6]. Model building was carried out iteratively using the Coot software [7]. Data collection and refinement statistics are given in Supplementary Table S2. Coordinates of the T_2R-TTL-TUB092 complex have been deposited at the Protein Data Bank (PDB) under accession number 5JVD.

CHEMISTRY

Synthesis

The synthesis of the proposed chalcones involved an aldol condensation between a phenone fused to a dioxolane ring (A ring) and the corresponding benzaldehyde (ring B). Firstly, we envisaged the synthesis of the unsubstituted chalcones at the α position of the α, β-unsaturated ketone (Scheme 1). Condensation of 14 [8] with differently substituted benzaldehydes (15a-d) in methanol in the presence of potassium hydroxyde [9] afforded the chalcones 5–8 in moderate yields (40–60%). Reduction of the nitrochalcone 8 employing tin chloride

(II) in the presence of 37% HCl [10] afforded the amino chalcone 9 in 68% yield. In almost all cases, a single isomer was detected. According to $J_{H\alpha\text{-}H\beta}$ values (~16 Hz) in the ^{1}H NMR spectra, these compounds were assigned as the *E*-isomers. Only for compound 7 a very minor isomer (around 5% as quantified by HPLC-MS compared to the major isomer) was also observed.

The synthesis of the α-methylchalcones started from the propiophenone 16 [11] (Scheme 1). Knoevenagel condensation between the propiophenone 16 and 3-hydroxy-4-methoxybenzaldehyde 15a by refluxing in the presence of piperidine and acetic acid, as described for related chalcones, [12] afforded the condensation product 10 in just 5% yield. By increasing the equivalents of piperidine and acetic acid, and by adding 4 Å molecular sieves to the reaction mixture, the chalcone 10 was obtained in 44% yield. In order to synthesize the corresponding aminochalcone 11, the 3-amino-4-methoxybenzaldehyde 15e was prepared as previously described [13]. Then condensation between 15e and the propiophenone 16 afforded the aminochalcone 11 in 43% yield. The *E*-configuration of the α-methylchalcones 10 and 11 was performed based on ^{1}H NMR ROESY experiments. It should be mentioned that only when the compounds were left in solution and light-exposed slow isomerization to the *Z*-isomer was observed, as described in similar analogues by photoisomerization [14].

The synthesis of the prodrugs was performed as described in Scheme 2. Reaction of 11 with the serine protected derivative 17 [15] in the presence of PyBOP and triethylamine afforded the conjugated derivative that was further treated with TFA to afford the L-Ser derivative 12. On the other hand, reaction of 11 with the dipeptide 18 [16] in the presence of HATU and DIPEA by microwave irradiation at 40 ºC, followed by deprotection with piperidine, afforded the L-Lys-L-Pro conjugate 13 (57% yield).

General chemistry procedures

Melting points were obtained on a Reichert-Jung Kofler apparatus and are uncorrected. The elemental analysis was performed with a Heraeus CHN-O-RAPID instrument. The elemental compositions of the compounds agreed to within ± 0.4% of the calculated values. For all the tested compounds, satisfactory elemental analysis was obtained supporting > 95% purity. Electrospray mass spectra were measured on a quadrupole mass spectrometer equipped with an electrospray source (Hewlett-Packard, LC/MS HP 1100). ^{1}H and ^{13}C NMR spectra were recorded on a Varian INOVA-300 operating at 299 MHz (^{1}H) and 75 MHz (^{13}C), respectively, a Varian INOVA-400 operating at 399 MHZ (^{1}H) and 99 MHz (^{13}C), respectively, and a INOVA SYSTEM-500 operating a 499 MHz (^{1}H) and 125 MHz (^{13}C), respectively.

Analytical TLC was performed on silica gel 60 F_{254} (Merck) precoated plates (0.2 mm). Spots were detected under UV light (254 nm) and/or charring with ninhydrin or phophomolibdic acid. Separations on silica gel were performed by preparative centrifugal circular thin-layer chromatography (CCTLC) on a Chromatotron[R] (Kiesegel 60 PF_{254} gipshaltig (Merck)), with layer thickness of 1 and 2 mm and flow rate of 4 or 8 mL/min, respectively. Flash column chromatography was performed in a Biotage Horizon instrument.

Microwave reactions were performed using the Biotage Initiator 2.0 single-mode cavity instrument from Biotage (Uppsala). Experiments were carried out in sealed microwave process vials utilizing the standard absorbance level (400 W maximum power). The temperature was measured with an IR sensor on the outside of the reaction vessel.

General procedure for the preparation of chalcones

To a solution of 1-(5′-methoxy-3′,4′-methylendioxyphenyl)ethanone (14) (1.0 mmol) in methanol (10 mL), a 50% (w/v) KOH aqueous solution (10 mL) and the corresponding benzaldehyde (15a–d) (1.2 mmol) were added and the reaction was stirred at room temperature for 24 h. Then, 1N HCl was added to facilitate the complete precipitation of the compounds. These were collected by filtration, and further purified by flash column chromatography.

(*E*)-3-(3′′-Hydroxy-4′′-methoxyphenyl)-1-(5′-methoxy-3′,4′-methylendioxyphenyl)prop-2-en-1-one (5)

Following the general procedure for the preparation of chalcones, reaction of 14 (40 mg, 0.21 mmol) and 3-hydroxy-4-methoxybenzaldehyde (15a) (38 mg, 0.25 mmol) afforded a residue that was purified by flash column chromatography (hexane/ethyl acetate) to yield 39 mg (57%) of 5 as a yellow solid. Mp: 162–164°C. MS (ES, positive mode): m/z 329 $(M+H)^+$. ^{1}H NMR (DMSO-d_6, 300 MHz) d: 3.84 (s, 3H, OCH_3-4′′), 3.94 (s, 3H, OCH_3-5′), 6.14 (s, 2H, CH_2), 7.99 (d, 1H, *J* = 8.4 Hz, Ar), 7.30 (dd, 1H, *J* = 8.4, 2.1 Hz, Ar), 7.35 (d, 1H, *J* = 2.1 Hz, Ar), 7.45 (d, 1H, *J* = 1.5 Hz, Ar), 7.47 (d, 1H, *J* = 1.5 Hz, Ar), 7.60 (d, 1H, *J* = 15.6 Hz, H-2), 7.70 (d, 1H, *J* = 15.6 Hz, H-3), 9.12 (br s, 1H, OH). ^{13}C NMR (DMSO-d_6, 100 MHz) d: 55.7 (OCH_3-4′′), 56.5 (OCH_3-5′), 102.4 (CH_2), 102.7, 109.1, 111.8, 115.0, 122.2, 127.8, 132.8, 139.2, 143.2, 146.6, 148.8, 150.2 (Ar), 119.3 (C-2), 144.2 (C-3), 186.8 (CO). Anal. calc. for ($C_{18}H_{16}O_6$): C, 65.85; H, 4.91. Found: C, 66.02; H, 4.71.

(*E*)-3-(3″-Fluoro-4″-methoxyphenyl)-1-(5′-methoxy-3′,4′-methylendioxyphenyl)prop-2-en-1-one (6)

Following the general procedure for the preparation of chalcones, reaction of 14 (40 mg, 0.21 mmol) and 3-fluoro-4-methoxybenzaldehyde (15b) (39 mg, 0.25 mmol) afforded a residue that was purified by flash column chromatography (hexane/ethyl acetate) to yield 43 mg (62%) of 6 as a yellow solid. Mp: 159-161 ºC. MS (ES, positive mode): m/z 331 $(M+H)^+$. ^{1}H NMR (DMSO-d_6, 300 MHz) d: 3.94 (s, 3H, OCH_3-4″), 3.99 (s, 3H, OCH_3-5′), 6.15 (s, 2H, CH_2), 7.22 (t, 1H, *J* = 8.7 Hz, Ar), 7.46 (d, 1H, *J* = 1.4 Hz, Ar), 7.50 (d, 1H, *J* = 1.7 Hz, Ar), 7.62 (dd, 1H, *J* = 7.5, 1.9 Hz, Ar), 7.64 (d, 1H, *J* = 15.5 Hz, H-2), 7.83 (d, 1H, *J* = 15.5 Hz, H-3), 7.96 (dd, 1H, *J* = 13.0, 2.0 Hz, Ar). ^{13}C NMR (DMSO-d_6, 100 MHz) d: 56.2 (OCH_3-4″), 56.5 (OCH_3-5′), 102.4 (CH_2), 102.9, 109.3, 113.7, 114.9, 127.3, 128.0, 139.4, 143.2, 148.8, 149.1, 150.7, 152.6 (Ar), 120.7 (C-2), 142.6 (C-3), 186.6 (CO). Anal. calc. for ($C_{18}H_{15}FO_5$): C, 65.45; H, 4.58. Found: C, 65.28; H, 4.39.

(*E/Z*)-1-(5′-Methoxy-3′,4′-methylendioxyphenyl)-3-(4″-methoxypyridin-3″-yl)prop-2-en-1-one (7)

Following the general procedure for the preparation of chalcones, reaction of 14 (40 mg, 0.21 mmol) and 6-methoxy-3-pyridinecarboxaldehyde (15c) (35 mg, 0.25 mmol) afforded a residue that was purified by flash column chromatography (hexane/ethyl acetate) to yield 31 mg (47%, two isomers *E/Z* ratio 95:5) of 7 as a yellow solid. MS (ES, positive mode): m/z 314 (M+H). ^{1}H NMR (DMSO-d_6, 300 MHz) d (*E* isomer): 3.91 (s, 3H, OCH_3-4″), 3.94 (s, 3H, OCH_3-5′), 6.15 (s, 2H, CH_2), 6.93 (d, 1H, *J* = 8.7 Hz, Ar), 7.47 (d, 1H, *J* = 1.1 Hz, Ar), 7.50 (d, 1H, *J* = 1.2 Hz, Ar), 7.72 (d, 1H, *J* = 15.6 Hz, H-2), 7.90 (d, 1H, *J* = 15.6 Hz, H-3), 8.39 (dd, 1H, *J* = 8.8, 2.5 Hz, Ar), 8.59 (d, 1H, *J* = 2.5 Hz, Ar). ^{13}C NMR (DMSO-d_6, 100 MHz) d (*E* isomer): 53.6 (OCH_3-4″), 56.5 (OCH_3-5′), 102.4 (CH_2), 102.9, 111.1, 120.9, 132.4, 137.8, 139.4, 143.2, 148.8, 149.5, 164.8, 186.6 (Ar), 124.6 (C-2), 140.3 (C-3), 186.8 (CO). Anal. calc. for ($C_{17}H_{15}NO_5$): C, 65.17; H, 4.83; N, 4.47. Found: C, 65.32; H, 4.75; N, 4.28.

(*E*)-3-(4″-Methoxy-3″-nitrophenyl)-1-(5′-methoxy-3′,4′-methylendioxyphenyl)prop-2-en-1-one (8)

Following the general procedure for the preparation of chalcones, reaction of 17 (100 mg, 0.51 mmol) and 4-methoxy-3-nitrobenzaldehyde (15d) (112 mg, 0.62 mmol) afforded a residue that was purified by flash column chromatography (hexane/ethyl acetate) to yield 90 mg (50%) of 8 as a yellow solid. Mp: 199-201 ºC. MS (ES, positive mode): m/z 358 $(M+H)^+$. ^{1}H NMR (DMSO-d_6, 300 MHz) d: 3.94 (s, 3H, OCH_3-4″), 3.99 (s, 3H, OCH_3-5′), 6.16 (s, 2H, CH_2), 7.44 (d, 1H, *J* = 8.9 Hz, Ar), 7.47 (d, 1H, *J* = 1.5 Hz, Ar), 7.55 (d, 1H, *J* = 1.5 Hz, Ar), 7.72 (d, 1H, *J* = 15.5 Hz, H-2), 7.95 (d, 1H, *J* = 15.5 Hz, H-3), 8.17 (dd, 1H, *J* = 8.9, 2.2 Hz, Ar), 8.51(d, 1H, *J* = 2.2 Hz, Ar). ^{13}C NMR (DMSO-d_6, 100 MHz) d: 56.5 (OCH_3-4″), 57.0 (OCH_3-5′), 102.5 (CH_2), 103.0, 109.4, 114.5, 124.4, 127.6, 132.3, 135.0, 139.5, 139.9, 143.3, 148.8, 152.9 (Ar), 122.0 (C-2), 141.2 (C-3), 186.5 (CO). Anal. calc. for ($C_{18}H_{15}NO_7$): C, 60.50; H, 4.23; N, 3.92. Found: C, 60.12; H, 4.36; N, 3.78.

(*E*)-3-(3″-Amino-4″-methoxyphenyl)-1-(5′-methoxy-3′,4′-methylendioxyphenyl)prop-2-en-1-one (9)

A solution of 8 (140 mg, 0.39 mmol), tin (II) chloride (371 mg, 1.96 mmol) and 37% HCl (0.20 mL) in ethanol/ethyl acetate (1:1) (6 mL) was stirred and refluxed for 2 hours. The cooled mixture was diluted with ethyl acetate (10 mL) and washed with saturated aqueous $NaHCO_3$ (10 mL). The organic layer was dried over $MgSO_4$, concentrated in vacuo and purified by flash chromatography (hexane/ethyl acetate) to yield 87 mg (68%) of 9 as yellow oil. MS (ES, positive mode): m/z 328 $(M+H)^+$. ^{1}H NMR (DMSO-d_6, 500 MHz) d: 3.83 (s, 3H, OCH_3-4″), 3.93 (s, 3H, OCH_3-5′), 4.81 (br s, 2H, NH_2), 6.14 (s, 2H, CH_2), 6.87 (d, 1H, *J* = 8.0 Hz, Ar), 7.11 (m, 2H, Ar, H-2), 7.40 (d, 1H, *J* = 1.0 Hz, Ar), 7.41 (d, 1H, *J* = 1.2 Hz, Ar), 7.57 (m, 2H, Ar, H-3). ^{13}C NMR (DMSO-d_6, 125 MHz) d: 55.5 (OCH_3-4″), 56.5 (OCH_3-5′), 102.4 (CH_2), 102.6, 109.1, 110.4, 112.9, 118.6, 124.5, 127.4, 132.9, 137.9, 139.1, 143.2, 148.9 (Ar), 119.2 (C-2), 144.8 (C-3), 186.8 (CO). Anal. calc. for ($C_{18}H_{17}NO_5$): C, 66.05; H, 5.23; N, 4.28. Found: C, 65.78; H, 5.12; N, 4.06.

General procedure for synthesis of α-methylchalcones

To a solution of the substituted propiophenone (1.0 mmol) and the corresponding benzaldehyde (0.5-1.0 mmol) in ethanol (4 mL), piperidine (1 mL), glacial acetic acid (0.5 mL) and 4 Å molecular sieves were added at room temperature. The mixture was stirred at 80 ºC under argon atmosphere for 16 h. Then, volatiles were removed in vacuo and the residue was purified as specified in each case.

(*E*)-3-(3″-Hydroxy-4″-methoxyphenyl)-1-(5′-methoxy-3′,4′-methylendioxyphenyl)-2-methylprop-2-en-1-one (10)

Following the general procedure for the synthesis of α-methylchalcones, reaction of 16 (100 mg, 0.48 mmol) and 3-hydroxy-4-methoxybenzaldehyde (18a) (37 mg, 0.24 mmol) afforded a residue that was purified by

CCTLC in the Chromatothron (hexane/ethyl acetate, 4:1) to yield 72 mg (88%) of 10 as a white solid. Mp 144-146 ºC.MS (ES, positive mode): m/z 343 $(M+H)^+$. ^{1}H NMR (DMSO-d_6, 500 MHz) d: d: 2.14 (s, 3H, CH_3), 3.80 (s, 3H, OCH_3-4″), 3.86 (s, 3H, OCH_3-5′), 6.12 (s, 2H, CH_2), 6.91 (s, 1H, Ar), 6.94 (d, 1H, *J* = 8.1 Hz, Ar), 6.97 (s, 1H, Ar), 7.00 (m, 3H, Ar, H-3),9.18 (br s, 1H, OH). ^{13}C NMR (DMSO-d_6, 125 MHz) d: 14.7 (CH_3), 56.0 (OCH_3-4″), 56.4 (OCH_3-5′), 102.2 (CH_2), 103.5, 110.0, 112.0, 116.7, 122.2, 128.1, 132.5, 138.1, 140.8, 146.3, 148.2, 148.4 (Ar), 133.6 (C-2), 142.8 (C-3), 197.3 (CO). Anal. calc. for ($C_{19}H_{18}O_6$): C, 66.66; H, 5.30. Found: C, 66.38; H, 5.09.

(*E/Z*)-3-(3″-Amino-4″-methoxyphenyl)-1-(5′-methoxy-3′,4′-methylendioxyphenyl)-2-methylprop-2-en-1-one (11)

Following the general procedure for the preparation of α-methylchalcones, reaction of the propiophenone 16 (256 mg, 1.70 mmol) and the aldehyde 15e (354 mg, 1.70 mmol) afforded a residue that was purified by flash column chromatography (hexane/ethyl acetate) to yield 250 mg (43%, two isomers *E/Z* ratio: 92:8) of 11 as yellow oil. MS (ES, positive mode): m/z 342 $(M+H)^+$. ^{1}H NMR (DMSO-d_6, 500 MHz) d (*E* isomer): 2.13 (d, 3H, *J* = 1.3 Hz, CH_3), 3.78 (s, 3H, OCH_3-4″), 3.84 (s, 3H, OCH_3-5′), 4.82 (br s, 2H, NH_2), 6.09 (s, 2H, CH_2), 6.70 (dd, 1H, *J* = 8.4, 2.1 Hz, Ar), 6.83 (d, 1H, *J* = 3.4 Hz, Ar), 6.84 (d, 1H, *J* = 3.1 Hz, Ar), 6.87 (d, 1H, *J* = 1.4 Hz, Ar), 6.97 (s, 1H, H-3), 6.98 (d, 1H, *J* = 1.4 Hz, Ar); ^{13}C NMR (DMSO-d_6, 125 MHz) d (*E* isomer): 22.8 (CH_3), 55.6 (OCH_3-4″), 56.1 (OCH_3-5′), 102.9 (CH_2), 103.1, 110.3, 110.5, 113.9, 116.9, 128.4, 133.5, 138.1, 138.4, 143.2, 147.6, 148.6 (Ar), 133.2 (C-2), 142.4 (C-3), 197.3 (CO). Anal. calc. for ($C_{19}H_{19}NO_5$): C, 66.85; H, 5.61; N, 4.10. Found: C, 67.02; H, 5.84; N, 4.00.

(*E/Z*)-3-(3″-Amino-L-seryl-4″-methoxyphenyl)-1-(5′-methoxy-3′,4′-methylendioxyphenyl)-2-methylprop-2-en-1-one (12)

To a solution of 11 (40 mg, 0.12 mmol) in $CHCl_3$ (1 mL), Boc-Ser(TBS)OH (17) (77 mg, 0.24 mmol), PyBOP (156 mg, 0.30 mmol) and triethylamine (46 µL, 0.36 mmol) were added and the mixture was stirred at room temperature for 24 h. Then, the crude was diluted with CH_2Cl_2 (20 mL) and washed with aqueous $NaHCO_3$ (10 mL) and brine (10 mL). The organic layer was dried over Na_2SO_4, filtered and evaporated to dryness. The residue was purified by CCTLC in the Chromatothron (hexane/ethyl acetate, 4:1) to yield 67 mg of the coupling product as a yellow oil. MS (ES, positive mode): m/z 665 $(M+Na)^+$. This product was dissolved in $CHCl_3$ (1 mL), and TFA (230 µL, 3.00 mmol) was added. The mixture was stirred at room temperature for 24 h. Volatiles were removed and the residue was purified by CCTLC in the Chromatothron (dichloromethane/methanol 20:1), yielding 30 mg (60% yield, two isomers, *E:Z* ratio 3:1) of 12 as a white solid. MS (ES, positive mode): m/z 429 $(M+H)^+$. ^{1}H NMR (DMSO-d_6, 500 MHz) d (*E* isomer): 2.17 (s, 3H, CH_3), 3.80 (m, 1H, CH), 3.83 (s, 3H, OCH_3-4″), 3.84 (s, 3H, OCH_3-5′), 3.90 (br s, 2H, NH_2), 3.99 (m, 2H, CH_2), 4.88 (t, 1H, *J* = 5.2 Hz, OH), 6.16 (s, 2H, OCH_2O), 6.81 (m, 2H, Ar), 6.89 (s, 1H, H-3), 6.97 (m, 2H, Ar), 8.10 (dd, 1H, *J* = 8.7, 2.3 Hz, Ar), 10.11 (br s, 1 H, NH). ^{13}C NMR (DMSO-d_6, 125 MHz) d (*E* isomer): 15.3 (CH_3), 55.9 (OCH_3), 59.1 (CH), 63.4 (CH_2), 102.8 (OCH_2O), 96.3, 110.6, 116.5, 116.8, 121.8, 127.4, 130.5, 130.6, 139.8, 143.5, 146.5, 150.6, (Ar), 135.7 (C-2), 143.6 (C-3), 171.8 (CONH), 205.2 (C-1). Anal. calc. for ($C_{22}H_{24}N_2O_7$): C, 61.68; H, 5.65; N, 6.54. Found: C, 61.56; H, 6.11; N, 6.33.

(*E/Z*)-3-(3″-amino-L-prolil-L-lisil-4″-methoxyphenyl)-1-(5′-methoxy-3′,4′-methylendioxyphenyl)-2-methylprop-2-en-1-one (13)

A microwave vial was charged with Fmoc-Lys(Fmoc)-Pro-OH (18) (206 mg, 0.30 mmol) and HATU (102 mg, 0.27 mmol) in anhydrous DMF (1 mL). Then DIPEA (61 µL, 0.35 mmol) and a solution of 11 (61 mg, 0.18 mmol) in DMF (1 mL) were added. The reaction mixture was heated at 40 ºC for 2 h. After cooling, the crude was diluted with CH_2Cl_2 (20 mL), acidified with 0.1 N citric acid (5 mL) and the organic phase was washed with $NaHCO_3$ (15 mL). After extracting with DCM (3x10 mL), the resulting organic layers were dried over Na_2SO_4 filtered, and evaporated to dryness. The residue was purified by CCTLC in the Chromatothron (hexane/ethyl acetate, 4:1) to yield 67 mg of the coupling product as yellow oil. MS (ES, positive mode): m/z 665 $(M+Na)^+$. This product was dissolved in CH_2Cl_2 (1 mL) and piperidine (50 µL, 0.52 mmol) was added. The reaction was stirred at room temperature for 2 h. Volatiles were removed in vacuo and the crude was purified by CCTLC in the Chromatothron (dichloromethane/7N NH_3 in methanol, 30:1) to yield 25 mg (88% yield, two isomers E:Z ratio 94:6) of 13 as a yellow oil. MS (ES, positive mode): m/z 567 $(M+H)^+$. ^{1}H NMR (DMSO-d_6, 500 MHz) d (*E* isomer): 1.34 (m, 4H, CH_2-γLys, CH_2-δLys), 1.53 (m, 2H, CH_2-β Lys), 1.91 (m, 4H, CH-bPro, CH-gPro), 2.16 (d, 3H, *J* = 1.3 Hz, CH_3), 2.56 (m, 2H, CH_2-ε Lys), 3.51 (m, 1H, CH-αLys), 3.66 (m, 2H, CH_2-dPro), 3.85 (s, 3H, OCH_3-4″), 3.88 (s, 3H, OCH_3-5′), 4.70 (m, 1H, CH-αProl), 6.11 (s, 2H, OCH_2O), 6.92 (d, J=1.4 Hz, 1H, Ar), 7.01 (d, J=1.4 Hz, 1H, Ar), 7.03 (s, 1H, H-3), 7.12 (d, J=8.7 Hz, 1H, Ar), 7.25 (dd, J=8.7, 2.2 Hz, 1H, Ar), 8.30 ((d, J=2.2 Hz, 1H, Ar), 9.35 (s, 1H, NHCO). ^{13}C NMR (DMSO-d_6, 125 MHz) d (*E* isomer):14.6 (CH_3), 21.6 (C-γLys), 22.3 (C-gPro), 27.7 (C-δLys), 28.6 (C-bPro), 32.5 (C-βLys), 41.3 (C-εLys),

46.6 (C-dPro), 52.0 (C-αLys), 56.0 (OCH_3-4″), 56.4 (OCH_3-5′), 58.5 (C-aPro), 102.2 (CH_2), 102.8, 103.4, 110.0, 127.4, 127.6, 115.3, 119.8, 127.5, 132.5, 138.2, 140.6, 148.2 (Ar), 132.5 (C-2), 142.8 (C-3), 170.1 (CO Pro), 196.9 (C-1), 198.2 (CO Lys). Anal. calc. for ($C_{30}H_{38}N_4O_7$. $2H_2O$): C, 59.79; H, 7.02; N, 9.30. Found: C, 59.62; H, 7.21; N, 9.57.

Scheme 1[(a)]

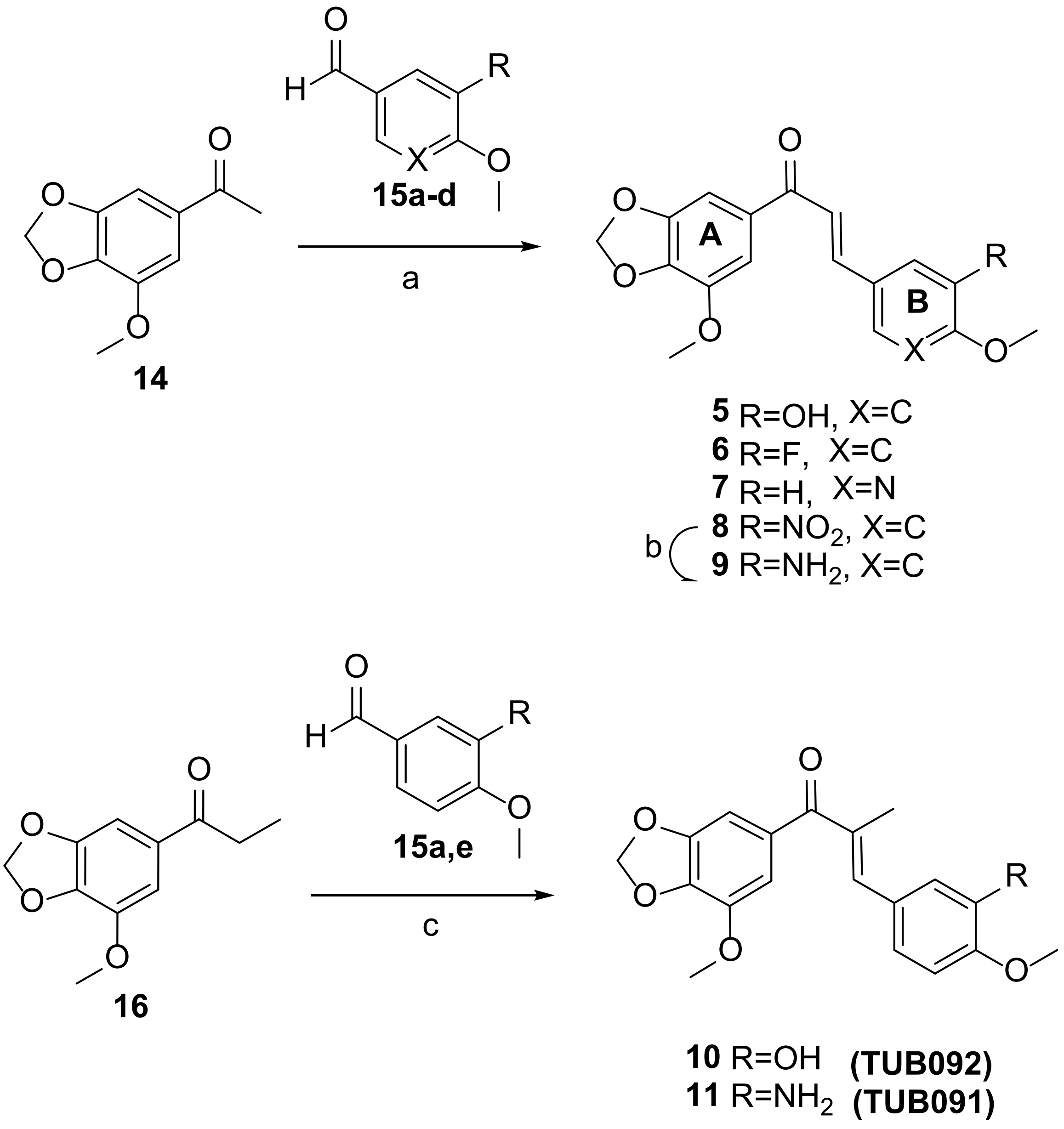

Reagents and conditions: (a) KOH 50%, MeOH, 25 ºC, 16 h; (b) $SnCl_2$, HCl, AcOEt, EtOH, 80 ºC, 2 h; (c) Piperidine, AcOH, EtOH, 4 Å molecular sieves, 80ºC, 16 h.

Scheme 2 [(a)]

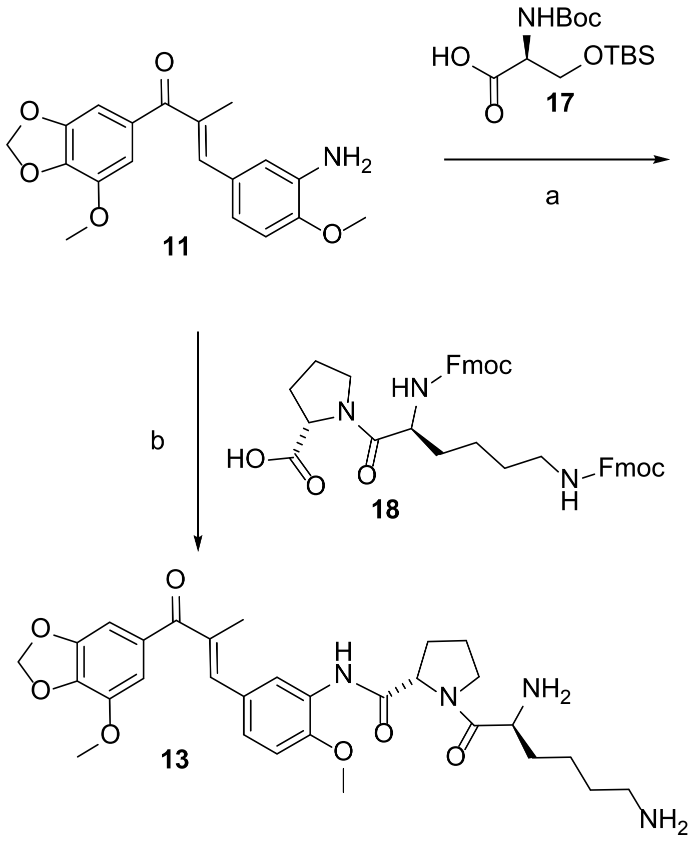

[(a)]Reagents and conditions: (a) (i) PyBOP, TEA, $CHCl_3$, 25 ºC, 72 h; (ii) TFA, $CHCl_3$, 25ºC, 24 h. (b) (i) HATU, DIPEA, anh.DMF, MW, 40ºC, 2 h; (ii) Piperidine, CH_2Cl_2, 25ºC, 2 h.

Supplementary Table S1: Growth-inhibitory activity of CA-4P and TUB091 in primary cells

Compound	IC_{50} (µM)	
	Hel	PBMC
CA-4P	0.0024 ± 0.0005	0.074 ± 0.019
TUB091	0.0018 ± 0.0009	0.037 ± 0.010

Growth-inhibitory activity is presented as IC_{50}, i.e. concentration that reduces cell growth by 50%. Hel: human embryonic lung fibroblasts, PBMC: peripheral blood mononuclear cells. Data are mean ± SD.

Supplementary Table S2: Crystallograpy: Data collection and refinement statistics

	T_2R-TTL-TUB092
Data collection[a]	
Space group	$P2_12_12_1$
Cell dimensions	
a, b, c (Å)	104.3, 156.3, 180.6
Resolution (Å)	49.5–2.39 (2.54–2.39)
R_{meas} (%)	13.3 (206.4)
R_{pim} (%)	
$CC_{1/2}$[b]	99.8 (36.1)
I/σI	11.6 (0.95)
Completeness (%)	99.6 (97.7)
Redundancy	7.0 (6.97)
Refinement	
Resolution (Å)	49.5–2.39
No. unique reflections	116285
R_{work}/R_{free} (%)	16.9/20.9
Average B-factors (Å^2)	
Complex	74.4
Solvent	61.2
Ligands (chain B/D)	51.5/62.7
Wilson B-factor	56.7
Root mean square deviation from ideality	
Bond length (Å)	0.005
Bond angles (°)	0.873
Ramachandran statistics[c]	
Favored regions (%)	98.3
Allowed regions (%)	1.7
Outliers (%)	0

[a]Highest shell statistics are in parentheses. [b]$CC_{1/2}$ = percentage of correlation between intensities from random half-datasets [17]. [c]As defined by MolProbity [18].

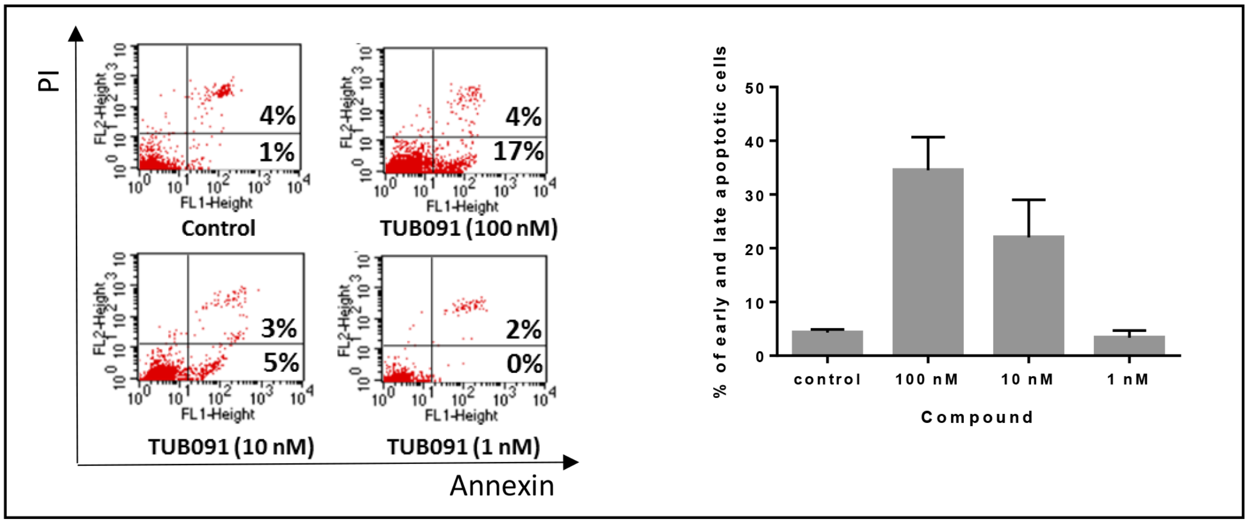

Supplementary Figure S1: Pro-apoptotic effect of TUB091. Annexin-PI staining of MDA-MB-231 cells that were treated for 24h with different concentrations of TUB091. Early apoptotic cells are characterized by high Annexin binding and low PI staining (lower, right quadrant), whereas late apoptotic (upper right quadrant) and necrotic cells (upper left quadrant) stain strongly for both Annexin and PI. Graph shows quantification of apoptotic cells (upper and lower right quadrant) as % of total. Values are expressed as mean ± SD.

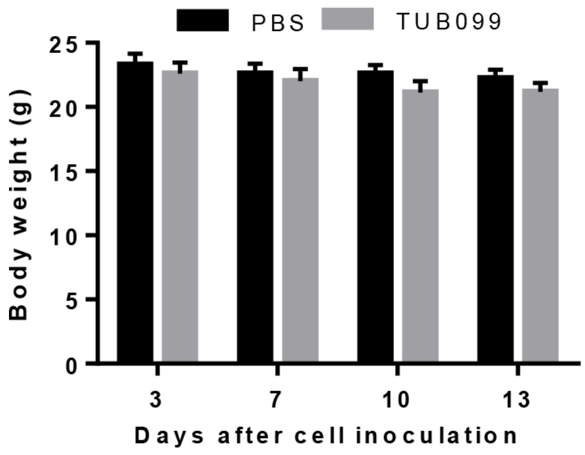

Supplementary Figure S2: Body weight of mice treated with 10 mg/kg of TUB099. Treatment with TUB099 (10 mg/kg) or vehicle was started 3 days after cell injection and continued for 5 consecutive days (days 3–7). Body weight was determined at regular time intervals.

REFERENCES

1. Liekens S, Gijsbers S, Vanstreels E, Daelemans D, De Clercq E, Hatse S. The nucleotide analog cidofovir suppresses basic fibroblast growth factor (FGF2) expression and signaling and induces apoptosis in FGF2-overexpressing endothelial cells. Mol Pharmacol. 2007; 71:695–703.
2. Prota AE, Bargsten K, Zurwerra D, Field JJ, Diaz JF, Altmann KH, Steinmetz MO. Molecular mechanism of action of microtubule-stabilizing anticancer agents. Science. 2013; 339:587–590.
3. Prota AE, Magiera MM, Kuijpers M, Bargsten K, Frey D, Wieser M, Jaussi R, Hoogenraad CC, Kammerer RA, Janke C, Steinmetz MO. Structural basis of tubulin tyrosination by tubulin tyrosine ligase. J Cell Biol. 2013; 200:259–270.
4. Prota AE, Danel F, Bachmann F, Bargsten K, Buey RM, Pohlmann J, Reinelt S, Lane H, Steinmetz MO. The novel microtubule-destabilizing drug BAL27862 binds to the colchicine site of tubulin with distinct effects on microtubule organization. J Mol Biol. 2014; 426:1848–1860.
5. Kabsch W. "XDS," Acta Crystallogr D Biol Crystallogr. 2010; 66:125.
6. Adams PD. ”PHENIX: a comprehensive Python-based system for macromolecular structure solution," Acta Crystallogr D Biol Crystallogr. 2010; 66: 213.
7. Emsley P, Cowtan K. "Coot: model-building tools for molecular graphics," Acta Crystallogr D Biol Crystallogr. 2004; 60:2126.
8. Messaoudi S, Tréguier B, Hamze A, Provot O, Peyrat JF, De Losada JR, Liu JM, Bignon J, Wdzieczak-Bakala J, Thoret S, Dubois J, Brion JD, Alami M. Isocombretastatins A versus Combretastatins A: The Forgotten isoCA-4 Isomer as a Highly Promising Cytotoxic and Antitubulin Agent. J Med Chem. 2009; 52:4538–4542.
9. Chiaradia LD, Dos Santos R, Vitor CE, Vieira AA, Leal PC, Nunes RJ, Calixto JB, Yunes RA. Synthesis and pharmacological activity of chalcones derived from 2,4,6-trimethoxyacetophenone in RAW 264.7 cells stimulated by LPS: Quantitative Structure–Activity Relationships. Bioorg Med Chem. 2008; 16:658–667.
10. Bonesi M, Loizzo MR, Statti GA, Michel S, Tillequin F, Menichini F. The synthesis and Angiotensin Converting Enzyme (ACE) inhibitory activity of chalcones and their pyrazole derivatives. Bioorg Med Chem Lett. 2010; 20:1990–1993.
11. Scheiders GE, Stevenson R. Structure and synthesis of (±)-wuweizisu C. J Org Chem. 1981; 46:2969–2971.
12. Ducki S, Rennison D, Woo M, Kendall A, Chabert JFD, McGown AT, Lawrence NJ. Combretastatin-like chalcones as inhibitors of microtubule polymerization. Part 1: synthesis and biological evaluation of antivascular activity. Bioorg Med Chem. 2009; 17:7698–7710.
13. Han X, Pradeep SND, Critchley K, Sheikh K, Bushby RJ, Evans SD. Supported bilayer lipid membrane arrays on photopatterned self-assembled monolayers. Chem Eur J. 2007; 13:7956–7964.
14. Perjési P, Takács M, Ösz E, Pintér Z, Vámos J, Takács-Novák K. In-solution and on-plate light-catalyzed E/Z isomerization of cyclic chalcone analogues. Lipophilicity of E- and Z-2-(X-benzylidene)-1-benzosuberones J Chromatogr Sci. 2005; 43:289–295.
15. Yoo D, Oh JS, Lee DW, Kim YG. Efficient synthesis of a configurationally stable L-serinal derivative. J Org Chem. 2003; 68:2979–2982.
16. García-Aparicio C, Bonache MC, Meester ID, San-Félix A, Balzarini J, Camarasa MJ, Velazquez S. Design and discovery of a novel dipeptidyl-peptidase IV (CD26)-based prodrug approach. J Med Chem. 2006; 49:5339–5351.
17. Karplus PA, Diederichs K. Linking crystallographic model and data quality. Science. 2012; 336:1030–1033.
18. Davis IW, Murray LW, Richardson JS, Richardson DC. MOLPROBITY: structure validation and all-atom contact analysis for nucleic acids and their complexes. Nucleic Acids Res. 2004; 32:W615–619.
